# Gene expression kinetics of *Exaiptasia pallida* innate immune response to *Vibrio parahaemolyticus* infection

**DOI:** 10.1186/s12864-020-07140-6

**Published:** 2020-11-09

**Authors:** François Seneca, David Davtian, Laurent Boyer, Dorota Czerucka

**Affiliations:** 1 Centre Scientifique de Monaco, 8 Quai Antoine 1er, 98000 Monaco, Monaco; 2 LIA ROPSE, Laboratoire International Associé Université Côte d’Azur - Centre Scientifique de Monaco, Monaco, Monaco; 3 Present Address: Division of Population Health & Genetics, Ninewells Hospital and Medical School, Dundee, DD19SY UK; 4 Université Côte d’Azur, C3M Inserm, U1065, 06204 Nice Cedex 3, France

**Keywords:** Innate immunity, Bacteria, Infection, Transcriptomics, Cnidaria

## Abstract

**Background:**

Recent sequencing projects on early-diverging metazoans such as cnidarians, have unveiled a rich innate immunity gene repertoire; however, little is known about immunity gene regulation in the host’s early response against marine bacterial pathogens over time. Here, we used RNA-seq on the sea anemone *Exaiptasia pallida* (Ep) strain CC7 as a model to depict the innate immune response during the onset of infection with the marine pathogenic bacteria *Vibrio parahaemolyticus* (Vp) clinical strain O3:K6, and lipopolysaccharides (LPS) exposure. Pairwise and time series analyses identified the genes responsive to infection as well as the kinetics of innate immune genes over time. Comparisons between the responses to live Vp and purified LPS was then performed.

**Results:**

Gene expression and functional analyses detected hundreds to thousands of genes responsive to the Vp infection after 1, 3, 6 and 12 h, including a few shared with the response to LPS. Our results bring to light the first indications that non-canonical cytoplasmic pattern recognition receptors (PRRs) such as NOD-like and RIG-I-like receptor homologs take part in the immune response of Ep. Over-expression of several members of the lectin-complement pathways in parallel with novel transmembrane and Ig containing ficolins (CniFLs) suggest an active defense against the pathogen. Although lacking typical Toll-like receptors (TLRs), Ep activates a TLR-like pathway including the up-regulation of MyD88, TRAF6, NF-κB and AP-1 genes, which are not induced under LPS treatment and therefore suggest an alternative ligand-to-PRR trigger. Two cytokine-dependent pathways involving Tumor necrosis factor receptors (TNFRs) and several other potential downstream signaling genes likely lead to inflammation and/or apoptosis. Finally, both the extrinsic and intrinsic apoptotic pathways were strongly supported by over-expression of effector and executioner genes.

**Conclusions:**

To our knowledge, this pioneering study is first to follow the kinetics of the innate immune response in a cnidarian during the onset of infection with a bacterial pathogen. Overall, our findings reveal the involvement of both novel immune gene candidates such as NLRs, RLRs and CniFLs, and previously identified TLR-like and apoptotic pathways in anthozoan innate immunity with a large amount of transcript-level evidence.

**Supplementary information:**

**Supplementary information** accompanies this paper at 10.1186/s12864-020-07140-6.

## Background

The immune system is an ancient and complex network that works to detect and defend an organism against pathogens, as well as to regulate the interactions between microbes and hosts. In vertebrates, the immune system consists of a two-fold mechanism: 1) the innate immunity which provides early protection to the host, and 2) the adaptive immune system capable of mounting a specific attack against invading pathogens and which displays memory. The current consensus is that invertebrates lack the components of the well characterized vertebrate adaptive immune system. However, with the recent proliferation of sequencing projects on early-diverging metazoans, the ancestral immune repertoire is clearly appearing more complex than previously suspected. As the sister group of bilaterians, cnidarians are the most early-diverging animals with true tissue layers and a gastro-vascular cavity, also known as the coelenteron. This game-changing internal feature is especially relevant to the evolution of innate immunity, as it marks the apparition of an internal location for interactions between host cells and microorganisms. Therefore, questions regarding the evolution of key bilaterian traits, such as pathogen and symbiont recognition, can be effectively explored using cnidarians. Indeed, cnidarians, including corals and anemones, have co-evolved since the Paleozoic era (570–245 Mya) with a multitude of unicellular algae, bacteria and viruses, and possess an innate immune system with an extensive list of genes homologous to those of the innate immunity of vertebrates [1–3]. However, when looking at the composition of specific immune pathways such as the TLR-to-NF-κB pathway across evolutionary divergent genera of cnidarians, key genes can be missing [2]. Therefore, comparative studies of the innate immune response in corals, *Hydra*, *Nematostella*, and *Exaiptasia* have the potential to help us understand how certain aspects of innate immunity evolved and may also point to alternative paths to activation of well conserved key factors of immunity.

Comparisons of the genomes of *Acropora* corals in symbiosis with algae, the aposymbiotic anemone *Nematostella vectensis* and the freshwater polyp *Hydra vulgaris* with those of other metazoans show increased complexity in gene families including Toll/Interleukin-1 Receptor- (TIR), NAIP/C2TA/HET-E/TP1- (NACHT), Tumor Necrosis Factor- (TNF) and Death-domains (DD) containing proteins [4–7]. In aiptasia’s (i.e. *Exaiptasia pallida* as renamed in [8]) transcriptome/genome ([9, 10], respectively), an active panel of immune-related genes is involved in the establishment of host-algae symbiosis [11], and the disruption of this symbiosis also known as bleaching [12], which warrants the question whether the same is true against pathogen invasion.

Overall, studies of innate immunity in cnidarians have focused on its evolutionary history [1, 4], its role in bacteria sensing and aging [13, 14], self−/non-self-recognition [15, 16], or within ecological contexts such as host-algae symbiosis [17], bleaching [18], wound healing [19] and diseases [20–23]. Although around twenty studies have investigated the innate immune response of cnidarian species to bacterial infection, only a few used the model *Exaiptasia* and none investigated the progress of the innate immune response over the onset of infection.

For example, *Exaiptasia* was used as a surrogate to better understand the diseases’ characteristics and interactions between coral pathogens such as *Serratia marcescens* (Sm) and *Vibrio* spp., and their hosts [24–26]. Later, Poole et al. [11] used quantitative real-time PCR to show that genes of the complement pathway positively respond to both colonization with *Symbiodinaceae* and infection with Sm in aiptasia. More recently, Roesel and Vollmer [27] used RNA-seq to compare the transcriptomic response of symbiotic and aposymbiotic (menthol-bleached) Ep to 24 h exposure to the bacterial pathogen *Vibrio coralliilyticus* (Vc). They found thousands of genes significantly differentially expressed suggesting roles of complement and coagulation cascades, NOD/Toll receptor signaling, and apoptosis in the innate immune response of Ep to *Vibrio* exposure.

Purified LPS - a Gram-negative specific endotoxin - has been used to induce a more controlled innate immune response in coral species ([28, 29] Williams et al. 2018) and in anemones [12, 30–32]. For example, in the coral *Acropora millepora*, the expression of a lectin homolog dubbed Millectin was found to bind to pathogens and be activated under both LPS and peptidoglycan treatments [29]. Palmer et al. [33] exposed three coral species to the endotoxin LPS and showed that different parameters of innate immunity such as prophenoloxidase activity and melanin concentration could vary greatly between species. Very recently, Connelly et al. [34] used RNA-seq to show that the innate immune responses to LPS treatment in two congeneric coral species were genotype-specific and only involved the up-regulation of 6 transcripts, confirming the difficulty of studying the innate immune response in corals. In *Exaiptasia*, LPS and peptidoglycan failed to trigger significant changes in gene expression [11]. Likewise, in 293-human cells, LPS did not activate a reconstituted TLR-to-NF-휅B pathway from both *Hydra* and *Nematostella*, although bacteria and flagellin did [32, 35].

Here, RNA-seq sequencing on clones of the symbiotic Ep strain CC7 was used to identify the innate immune response of the host to bacterial infection. To challenge the innate immune system of *Exaiptasia*, the Gram-negative halophilic *Vibrio parahaemolyticus* (Vp) was chosen because marine zoonotic pathogens such as Vp strain O3:K6 can have disastrous consequences for human populations. Indeed, Vp is the leading cause of marine-borne illnesses worldwide [36] causing gastroenteritis via the consumption of contaminated seafood [37, 38]. On the other hand, environmental strains naturally occur in the mucus of coral species [39, 40], which make Vp a member of the rich bacterial community associated with cnidarians. This study focuses on the gene regulation at each time point and the gene expression kinetics of immune-related genes in response to 1, 3, 6 and 12-h exposures to the pathogen, and contact with LPS for 3, 6 and 12 h. Our results improve our understanding of innate immunity through the measurement of gene activity in response to infection in one of the most basally ancestral tissue-forming animals and highlights several fascinating gene targets for future functional studies.

## Results

### Physiological response to infection

**Fig. 1:**
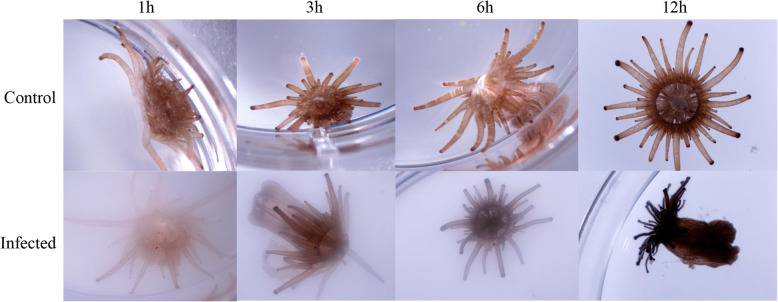
Photographs of several *Exaiptasia pallida* individuals which show the progressive shriveling of tentacles over 12 h of exposure to Vp. Photos were taken using a stereomicroscope mounted with a digital camera under 0.8X magnification after 1, 3, 6, and 12 h of exposure to experimental conditions: FSW for the controls and 10^8^ cfu/ml of Vp for the infection treatments. The cloudiness on the bottom row results from the Vp in solution

In order to study Ep’s innate immune response to Vp infection over time, we first visually monitored the response of anemones over 48 h. Animals removed from the treatment medium after 12 or 24 h, and subsequently left in filtered seawater (FSW) for another 36 or 24 h, recovered their original appearances after the temporary assault (Supplementary Figure 1). Physiologically, Ep’s response to infection consists of condensed tentacles (Fig. 1), increased mucus production, shriveling and cell detachment. Under the microscope, Vp in contact with the host can be seen swarming all over the outward surface of the anemones, which shows signs of tissue damage and invasion at 12 and 24 h post-inoculation. After 24 h in the infectious medium some mortality among Ep specimens was observed. To investigate the early innate immune response that allows Ep to defend itself against Vp’s attack, we focused our gene expression analyses on the first 12 h of infection, when all anemone specimens remained alive.

### Transcriptome assembly and annotation

Sample libraries - from 8 to 28 million raw reads across 23 samples (one sample failed quality control) - included both control and infected anemones at all four time points, and were assembled into 321,413 contiguous sequences or contigs, resulting in the holobiont de novo transcriptome (i.e. genome-free assembly). This transcriptome assembly was then aligned to the peptide sequences predicted from the aposymbiotic (deprived of algae) Ep strain CC7 genome from the Pringle laboratory at Stanford University [10] to generate our *Exaiptasia*-specific reference transcriptome. This alignment identified 132,179 contigs (41% of the holobiont transcriptome) specific to Ep using BLASTX, which were then exclusively used in the rest of the analyses. Our reference transcriptome includes contigs corresponding to non-overlapping fragments of the same transcript. Thus, contigs represent both complete and partial gene sequences with 1 to 47 ‘isoforms’ per gene (as determined by the TRINITY assembler). The length of contigs ranges from 201 to 49,073 bp with an average length of 950 bp. On average 74% of reads per sample align to the reference transcriptome (number of reads per sample mapping to the reference transcriptome are given in Supplementary Table 1).

Among the reference transcriptome contigs, 130,037 (98%) retrieve a match from the NCBI non-redundant database (e-value < 10^− 3^ and % similarity > 35), 84,369 (64%) were annotated with Gene Ontology (GO) terms, 17,192 with KEGG enzyme codes, and 61,980 with InterPro IDs. GO annotations for the whole reference transcriptome resulted in 9240 unique GO terms. Among these, 2761 belong to 82 different “immune_class” categories of the CateGOrizer tool (Supplementary Figure 2).

### Transcriptome-wide trends

RNA-seq data was produced for 32 anemones corresponding to three biological replicates at each time point and for each experimental group: controls and infected anemones at the four time points (minus one control anemone which did not pass quality control), and anemones exposed to LPS only for 3, 6, and 12 h. The data was analyzed using two methods: 1) a pairwise comparison of gene expression levels between treated and control anemones at each time point, and 2) a time series analysis of the changes in gene expression over time for each experimental group.

The general trends primarily revealed from the pairwise gene expression analyses between control and infected samples at each of the four time points show that: 1) more genes are up-regulated than down-regulated throughout the host response to infection; 2) the number of regulated genes increases with time; and 3) the regulatory activity in response to the infection over 12 h ranges from ≤1 to 3% of the whole transcriptome.

The proportion of the aiptasia-specific DECs within the holobiont (i.e. the host and its symbionts) response ranges from 70 to 83% across up−/down-regulated DECs at the four time points of the experiment (Table 1). The numbers of up- and down-regulated DECs are similar after 1-h post-infection, but the percentage of up-regulated DECs dramatically increased to 81% of the Ep gene activity after 3 h. This relationship weakens with time but remains high (73 and 63% of DECs up-regulated) over the next two time points. Among the aiptasia-specific DECs, 2% of up- and < 1% of down-regulated DECs represented the immune related GO terms. To complement the list of DECs potentially involved in the innate immune response of Ep, more permissive lists were created to include genes associated with the recognition of the pathogen, the transduction of a defensive signal and the degradation of the invader. Thus, 2–8% of the aiptasia-specific up-regulated DECs represented the receptor activity, signal transduction and peptidase activity GO classes, while the same GO classes were represented by 2–6% of the down-regulated DECs. The up- and down-regulated genesets were then compared to each other across time points to identify DECs unique or shared between the different time points via Venn diagrams (Supplementary Figure 3). These Venn diagrams show that: 1) there are more genes unique to each time point than genes detected twice or several times; 2) there are very few genes continuously regulated during 12 h of infection; and 3) later time points share more genes. These trends suggest a progression in the host response even though anemones were kept in the same infectious medium over 12 h. As Vp cells settle down and anemones concentrate them from the medium, host-pathogen physical interaction likely decreases with time, while signal transduction from first pathogen contact to effector activation progresses.

**Table 1:** Proportions of DECs of interest across the response to Vp infection - Number of DECs (robust Exact test at FDR < 0.05) detected at each time point for the holobiont transcriptome, the transcriptome of aiptasia alone, and genes belonging to the four GO classes of interest in this study: 'immune response', 'receptor activity', 'signal transduction', and 'peptidase activity'. The percentage of DECs belonging to the aiptasia-specific response is given in parentheses next to each DECs number

Number of DECs at:	1 h post infection	3 h post infection	6 h post infection	12 h post infection
**Holobiont-wide transcriptomic activity**				
Up-regulated	669	2198	2445	3754
Down-regulated	570	536	785	2025
**Aiptasia-specific transcriptomic activity (% of holobiont’s activity)**				
Up-regulated	509 (76%)	1697 (77%)	1764 (72%)	2614 (70%)
Down-regulated	418 (73%)	414 (77%)	656 (83%)	1584 (78%)
**DECs representing Ap’s immune response (% of Ap’s activity)**				
Up-regulated	12 (2%)	34 (2%)	34 (2%)	41 (2%)
Down-regulated	0 (< 1%)	1 (< 1%)	4 (< 1%)	10 (< 1%)
**DECs representing Ap’s receptor activity**				
Up-regulated	42 (8%)	40 (2%)	49 (3%)	66 (2.5%)
Down-regulated	15 (4%)	24 (6%)	22 (3%)	50 (3%)
**DECs representing Ap’s signal transduction**				
Up-regulated	31 (6%)	71 (4%)	94 (5%)	128 (5%)
Down-regulated	18 (4%)	11 (3%)	24 (4%)	67 (4%)
**DECs representing Ap’s peptidase activity**				
Up-regulated	17 (3%)	80 (5%)	120 (7%)	196 (7.5%)
Down-regulated	10 (2%)	12 (3%)	15 (2%)	43 (3%)

### Progression of innate immunity over 12 h

**Fig. 2:**
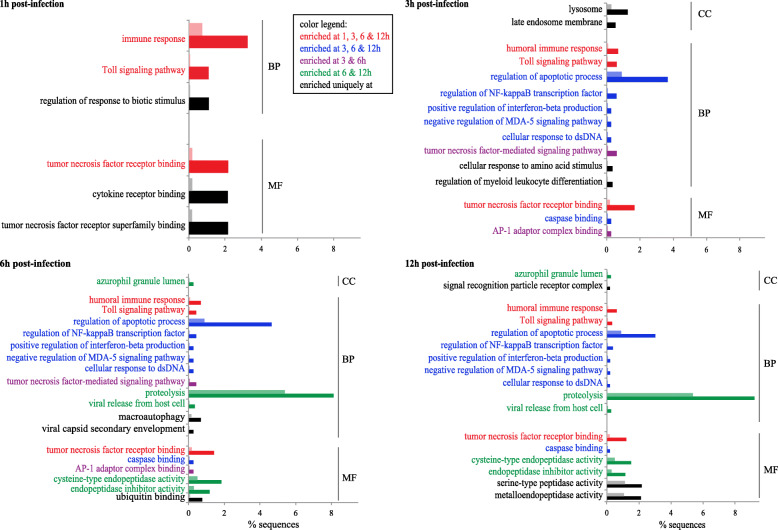
Immune response-related GO classes significantly enriched (FET; FDR < 0.05) in the transcriptomes of Vp treated samples compared to controls after 1, 3, 6, and 12 h post-infection. Results are illustrated by horizontal bars corresponding to the percentage of sequences representing each GO class. The lighter bars illustrate the percentage of sequences for each GO class in the reference transcriptome geneset, while the vibrant bars are the percentage of sequences in the significantly regulated geneset under infection conditions. The evolution of the immune response through time can be followed across the four panels for the three general GO categories: BP: biological processes, MF: molecular functions, and CC: cellular components. A color code is used to help distinguish the categories unique or shared between time points

The Fisher’s Exact Test (FET) performed on the up- and down-regulated lists of DECs detected at each time point highlights many significantly enriched GO classes unrelated to the immune response of Ep, but rather its maintenance of homeostasis and the energetic cost of its defense against pathogenic invasion. Although those aspects of Ep’s response to bacterial infection are of interest as well, we chose to focus on innate immunity. Therefore, Fig. 2 only shows the results of enrichment analyses performed on up-regulated DECs and GO categories related to different aspects of the immune response. The enrichment analyses’ results on down-regulated DECs are shown in Supplementary Figure 4.

The results of the enrichment analyses strongly support an innate immune response activity already detectable after 1 h and involving up-regulated genes linked to the ‘Toll signaling pathway’ biological process (GO_BP) and ‘TNFR-binding’ molecular function (GO_MF) represented throughout the experiment (Fig. 2; FET: FDR < 0.05; Supplementary Table 2 for statistical values). As the immune response progresses, the ‘regulation of apoptotic process’ becomes evident after 3 h, and remains apparent through the rest of the experiment. Among the genes indicating apoptotic activity, some belong to the ‘regulation of: NF-κB transcription factor, interferon-beta production, and MDA-5 signaling pathway’ GO_BP - as well as the GO_MF ‘caspase binding’ highlighted at 3, 6, and 12 h post-infection. The role of the TNF pathway in the immune response of Ep gathers even more support at 3 and 6 h, with the enrichment of ‘TNF-mediated signaling’. The ‘binding to AP-1 adaptor complex’ is highlighted at these two time points as well. At 6 h, ‘activity of endopeptidase’ and ‘endopeptidase inhibitor’ was detected for the first time and was evident again at 12 h. Finally, after 12 h of infection, while activity within TNF receptor, apoptosis, and proteolysis pathways is still strong, additional peptidase activities join in the host response. In summary, these results reveal a noticeable chronology in the immune activity of Ep against the pathogen, starting with the involvement of TNFRs, then the activation of apoptosis followed by proteolysis. The genes supporting these functional categories, as well as DECs without GO annotations but containing immunity-related conserved domains such as DD, Ig, lectin, leucine-rich repeat (LRR), NACHT, scavenger receptor (SR), TIR, and TNF represent an interesting source of novel innate immunity candidates.

### Profiling of seven gene expression clusters

**Fig. 3:**
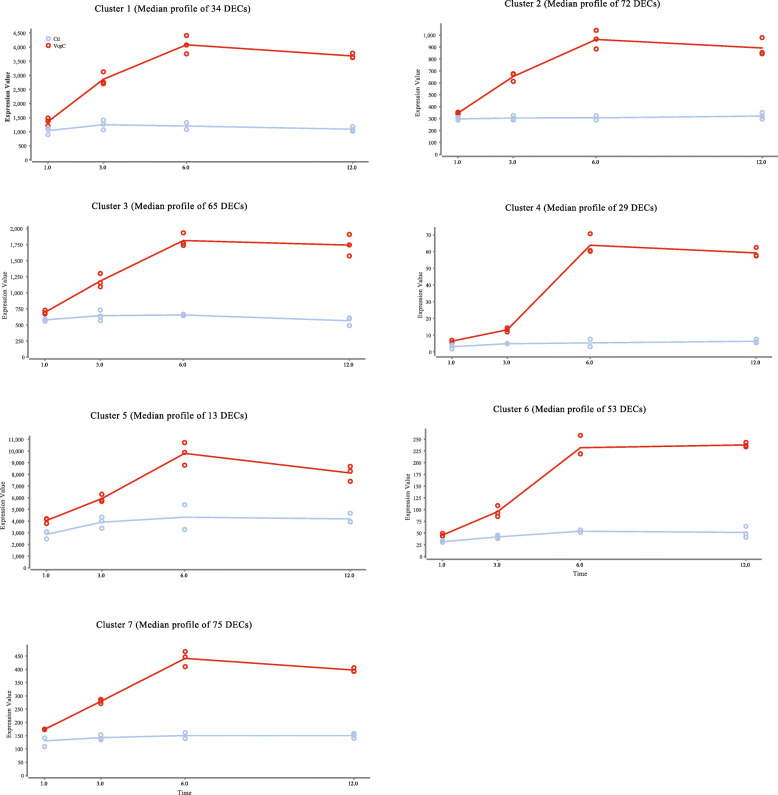
Median expression profiles of seven contig clusters representing DECs with similar kinetics over 1, 3, 6 and 12 h. The expression profiles under infection conditions are in red, and the controls in blue. The median normalized expression for contig clusters per sample is given on the y-axis

The time series analysis comparing the changes in expression of DECs responding to Vp infection over time identified seven profiles (Fig. 3). These profiles consist of plots showing the median expression levels for groups or clusters of DECs ranging from 13 to 75 contigs. Expression profiles diverge based on the changes with time but also the overall levels of expression of the DEC clusters. For example, cluster 1, 2 and 3 show very similar trends over time but display widely different median levels of expression. The overall trends across the seven profiles seem to mainly diverge at the 3 and 12-h time points, while median expression levels are specific to each cluster. When looking at the genes represented in each cluster, annotations with relevance to innate immunity were noticed in each cluster. For example, Cluster 1 includes DECs homologous to transcription factor MafB-like, NF-κB, and ETS-related transcription factor Elf-3; Cluster 3: TNFR superfamily member 27, TRAF 2-like, NF-κB p100 subunit-like, complement C2-like, and complement factor B precursor; and Cluster 6: bcl-2-like protein 1, TNFR superfamily member 1A-like, TRAF 3, Cell death protein 3, WD repeat domain phosphoinositide-interacting protein 2-like, Protein NLRC5, and Macrophage scavenger receptor types I and II. A full list of the DECs detected by the time series analysis and organized by cluster is given in Supplementary Table 3.

### Identification of innate immunity genes

**Fig. 4:**
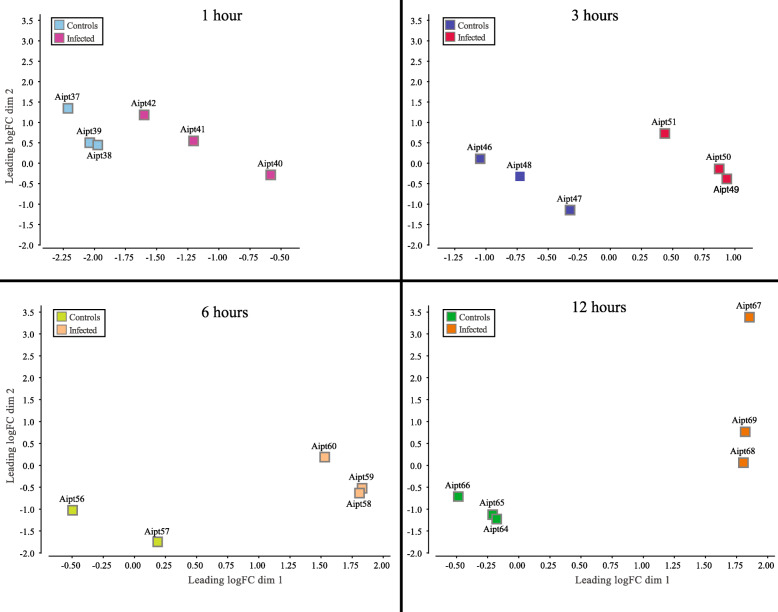
Multidimensional scaling (MDS) plots of transcriptomic responses represented in log Fold Change (logFC) show differences among samples with the treatments separated primarily on the dimension 1 and the influence of time on the dimension 2

The pairwise gene expression analyses detected hundreds to thousands of DECs at each time point, mounting to a total of 6842 unique DECs responding to the infection (Supplementary Tables 4 and 5). These strong effects of the infection on the transcriptome of *Exaiptasia* are illustrated in Fig. 4. Firstly, the effects of infection are clearly separating on dimension 1 with controls and infected samples distinctly separated at each time point. Secondly, this separation between treatments becomes greater and greater over time also on dimension 1. Bacterial infection is progressive, therefore as the pathogens invade the anemones’ tissue, the transcriptomic activity in the host, reflected here by the number of DECs detected, increases over time. In addition, a subtler influence of time is illustrated by the separation between the treatments on dimension 2. This separation is initially detectable at the 3-h time point, and increases obviously at the 12-h time point (Fig. 4). The fold changes illustrated in Fig. 4 are provided in Supplementary Table 6, which follows the organization chosen by Zhang et al. [41] in a relevant study that analyzed the transcriptomic response of the amphioxus - *Branchiostoma belcheri* - to Vp.

Among the most regulated and well supported (i.e. showing high similarity) DECs (Supplementary Table 6), several gene families including coagulation factor, lectin, NLR, SR, interferon regulatory factor (IRF), and TNFR-associated factors were highly represented. For example, the most highly regulated immune related DECs at 1-h post-infection are the IRF1-like sequences (FC = 795.7, Robust Exact Test - FDR = 6.52E^− 3^; and FC = − 98.1, Robust Exact Test - FDR = 4.09E^− 2^), which are accompanied by isoforms up-regulated at all three other time points (Supplementary Table 4). At 3 h post-infection, the TNF receptor-associated factor 5 (TRAF5) -like DEC is down-regulated 230.6 times (Robust Exact Test, FDR = 1.79E^− 2^), but isoforms are up-regulated at 3, 6 and 12 h (Supplementary Table 4). From the same gene family, but after 6 h post-infection, the TRAF4 DEC shows the highest up-regulation by a fold change of 488.2 (Robust Exact Test, FDR = 1.04E^− 3^). In the absence of TLRs in the genome of aiptasia, genes containing SR, Lectin, or LRR domains belonging to the macrophage scavenger receptor (MSR), lectin or NLR families potentially represent the pathogen recognition capacity in Ep, discussed further below. Furthermore, ligands and receptors of the TNF family suggest the induction of potential cytokine dependent pathways possibly connecting to an apoptosis-like pathway reported below.

### Apoptotic gene activity

Similar to the innate immunity genes illustrated in Supplementary Table 6, highly regulated genes associated with apoptosis are shown in Supplementary Table 7. DECs likely involved in the apoptotic process were already detected after 1-h post-infection and further progression of the apoptotic signal was observed at each following time point. For example, at the 3-h time point, a homolog of ‘v-ets erythroblastosis virus E26 oncogene’ is up-regulated 249.4 times (FDR = 3.68E^− 5^) and was still up-regulated at 6 and 12 h (Supplementary Table 5). A homolog of the key apoptotic executor – CASP3 – is detected as the overall most up-regulated DEC of this analysis, with a fold change of 1250.5 after 6 h post-infection (Robust Exact Test - FDR = 3.06E^− 17^), in addition to isoforms also detected at 3 and 12 h (Supplementary Table 5). Briefly, the genes in Supplementary Table 7 suggest that both the extrinsic and intrinsic apoptotic pathways are playing a role in the anemone’s response. The former is likely represented by caspase 8 (CASP8), Ced3 (CASP9) and CASP3, and the latter by mitochondria-associated genes such as Bcl-2, Bcl-2-associated X protein (Bax), and Bcl-2 antagonist/killer (Bak), among others. DECs representing the Ced3 gene take part in the expression profiles of clusters 6 and 7, while DECs for Bcl-2 are represented by clusters 1, 3 and 6 (Fig. 3). In addition, the apoptotic initiator Apaf-1 is also represented in the expression profiles of clusters 2 and 7 (Fig. 3).

### Lack of TLR-like pathway activation under LPS

**Fig. 5:**
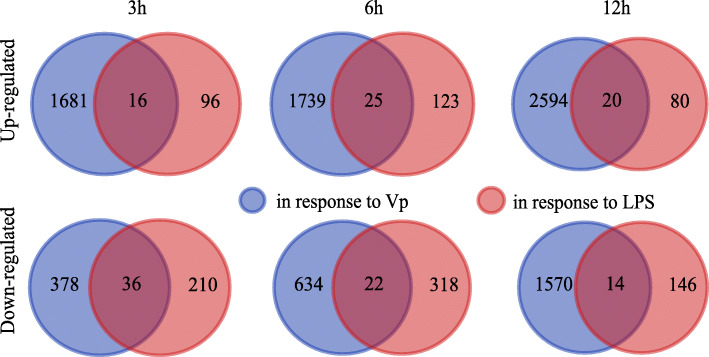
Venn diagrams comparing the up- and down-regulated DECs detected in the pairwise analyses, which evaluate Vp-infected versus LPS-treated anemones and controls. The numbers of DECs detected in response to Vp infection are in blue, while the DECs responding to LPS treatments are in red. The intersections between colors show the number of DECs in common to both treatments

In order to identify the genes potentially responding to the pathogen-associated molecular pattern molecule (PAMP) LPS, lists of DECs produced as a result of 3, 6 and 12 h of LPS treatment were compared to the DECs responding to the Vp infection at the same time points. Only a few DECs were found in common to both treatments (the intersections in Fig. 5), and neither TLR homologs nor orthologs of the *Hydra*’s HyLRR-2 and HyTRR-1 genes [42] were identified in the Ep’s responses to LPS or Vp (Supplementary Table 8 versus Supplementary Tables 4 and 5, respectively). Based on these comparisons (Fig. 5), a TLR-independent TLR-like pathway possibly represented by homologs of MyD88, TRAF, TBK1, NIK, IRF, NF-κB and AP-1, is activated under Vp infection but not LPS exposure. On the other hand, exposure to LPS induces TNF family homologs such as TNFR superfamily member 16 (TNFRSF-mb16) and LPS-induced TNFα factor up-regulation after 6 and 12 h, respectively. Moreover, searching for potential receptors of PAMP among the DECs triggered by LPS reveals an active regulation of many genes potentially involved in sensorial pathways including G-protein coupled- and various neuronal receptors (Supplementary Table 8).

## Discussion

This time series RNA-seq experiment was conducted to investigate the molecular innate immune response of the symbiotic *Exaiptasia pallida* strain CC7 to *Vibrio parahaemolyticus* strain O3:K6 infection over 12 h. Analyses focus on three main aspects of the innate immune response of the host: 1) the genes differentially expressed in infected anemones, 2) the gene expression changes over the onset of infection, and 3) the comparison between the responses to Vp and exposure to the endotoxins LPS. Punctual pairwise gene expression and time series analyses of *Exaiptasia*’s transcriptomic response to the virulent pathogen Vp identified 6842 differentially expressed contigs responsive to bacterial infection across four time points. Below we discuss pathway level kinetics that contribute pertinent information to previously described innate immune gene families as well as novel innate immune gene candidates.

### Physiological response to bacterial challenge

Ep exposed to the clinical O3:K6 Vp strain at 10^8^ cfu/ml starts to show mortality after 24 h. Similar to this cross-pathogenicity, another study using the gram-negative human pathogen - *Serratia marcescens -* at the same concentration showed that Ep has a survival rate of 70% after 24 h [26]. Our observations of Ep’s physiological response to Vp also concur with the descriptions of the same species infected with the coral pathogens Vc and *V. shiloi* [24]. Although darkening of the tissues during those infections was linked to melanin production, our search for evidence of melanin biosynthesis at the transcript level failed to identify homologs in this dataset. Anemones exposed to Vp show darkening of their tissues, retraction of tentacles, and mortality after 24 h post-inoculation. Thus, our results suggest that the clinical Vp strain O3:K6 responsible for gastroenteritis in humans remains pathogenic in saltwater at 27 degrees Celsius, and can successfully infect and start killing aiptasia anemones after 24 h. Brown and Rodriguez-Lanetty [43] determined that an exposure to 10^8^ cfu/ml of Vc at 30 °C for 3 days was a sub-lethal treatment from which 100% of Ep exposed could recover. A similar result was observed here but after only 12 h of exposure to 10^8^ cfu/ml of Vp at 27 °C. Based on these results, Ep likely possesses an innate immune system capable of efficiently fighting back seriously challenging and varied pathogenic assaults.

### Functional support for non-canonical cytoplasmic PRRs

Within cells, intracellular PRRs detect pathogens and are critical in the response to microbial components in the cytosol. Here, homologs of NLRs, NLRC-like genes were significantly regulated at each time point (Supplementary Table 4) and include up-regulated sequences with NACHT-like domains and LRR as well as a down-regulated sequence with trans-membrane domains (TMD). The NLRs are cytoplasmic proteins, which recognize bacterial peptidoglycans and trigger proinflammatory and antimicrobial immune response. Among the NLR family, the NLR family CARD domain containing protein 4 (NLRC4) is the best-known mammalian member capable of triggering the inflammasome upon detection of microbial ligands [44]. Interestingly, a large and complex NLR repertoire is present in early-diverging metazoans, including cnidarians, which exhibit an especially rich family of NACHT/NB-ARC containing genes in comparison with many later-diverging metazoans [6, 7]. In actinarians, the diverse repertoire of NLR-like genes and their novel domain architecture has been hypothesized to perform novel roles in immune signaling and/or pathogen recognition, which are yet to be described [10, 45]. Among the 78 NLRC contigs regulated during Ep’s infection, some contain LRR motifs (Supplementary Table 4), suggesting a capacity to bind to PAMP. These genes warrant further functional investigations to determine whether they play a role in the recognition of pathogens within the cells.

Additional genes were identified as potential cytoplasmic PRRs, including interferon-induced helicase C domain-containing protein 1 (IFIH1) and RIG-I-like receptor 3 (RLR-3), which both contain the RIG domain of the RLRs and play a role in the intracellular recognition of viruses. In total eight IFIH1 sequences and three RLR-3 sequences were detected as up-regulated at 3, 6 and 12 h (Supplementary Table 4). As the pathogen Vp acquired several new genomic islands through horizontal transfer, which contain phage-encoded virulence factors [46], it is possible that the Ep genes mentioned above are induced by viral-like genetic material brought into the host cells by invading Vp. To our knowledge, this is the first report of infection-induced RLR in a cnidarian.

### Involvement of the lectin-complement pathway

In the response to Vp infection, we found that anemones up-regulated Ficolin-1 homologs containing TMD and Ig (unlike bilaterian ficolins [45];) at 3, 6, and 12 h (Supplementary Table 4), as well as several components of the lectin-complement pathway. For example, although no regulation of MASP was detected, complement component C2, C3 and C4 were up-regulated over time. Moreover, aiptasia’s factor B (Ep_Bf-1) of the alternative pathway was up-regulated in the infected anemones at 3, 6 and 12 h (Supplementary Table 4). Poole et al. [11] used quantitative real-time PCR to show up-regulation of both Ep’s Bf-1 and MASP genes in response to 24-h exposure to low and high concentrations of the pathogen *Serratia marcescens*, and concluded that concentration of microbes, and symbiotic state influence complement gene expression. The co-expression of TMD/Ig-containing Ficolin-1 and genes of the lectin-complement pathways detected here supports a potential role of CniFLs in the initial recognition of pathogens [10], and highlights both the lectin and alternative pathways active roles in the defense of anthozoans against pathogenic bacteria. Nevertheless, it remains to be demonstrated whether the CniFLs bind to certain PAMP and can trigger the lectin-complement pathway, potentially stimulating phagocytes in aiptasia.

Several other lectins were detected during the response to Vp infection. For example, L-rhamnose-binding lectins (Ep_RBLs) contigs, homologs of collectin-12 (colec12), and a single C-type lectin LRR-containing sequence were up-regulated (Supplementary Table 4). RBL interact with various types of bacteria in fish and invertebrates to induce proinflammatory cytokines such as IL-1β, TNFα and IL-8, and enhance macrophage phagocytosis [47]. The scavenger receptor lectin-like family is rich and diverse in anthozoans [31], plays an important role in host-symbiont interactions during onset of symbiosis [17], and can bind to pathogens [48]. Our results highlight the diversity of lectins potentially involved in the recognition, potential activation of phagocytosis, and proinflammatory signaling upon contact with a pathogen in aiptasia.

### TLR-like pathway without TLR

Here, neither homologs of canonical vertebrate TLR or IL-1R, nor orthologs of the *Hydra*’s HyTRR and HyLRR genes [42] were identified in the Ep transcriptomic responses. TLR and IL-1R-like homologs have been identified in several other anthozoans [49], however many of those genes lack the ectodomain LRR and only possess TMD- and TIR-domains [10]. Aiptasia’s genome possesses homologs of the major PRR types including NLRs, RLRs, and C-type lectin receptors (CLRs) but lack TLRs. However, TLR-to-NF-κB pathway components are definitely present in Ep, as well as in *A. digitifera* and *O. faveolata* corals, *Hydra* and *Nematostella* (reviewed in [2]). Several of these genes include homologs with well conserved domains such as MyD88, TRAF6, IRFs, AP-1, and NF-κB, and were positively responsive to Vp infection (Supplementary Table 4). Functional conservation of the TLR-to-NF-κB was confirmed in *Nematostella* [32] and *O. faveolata* [50]. Furthermore, in *Hydra*, MyD88-deficient polyps were more susceptible to infection with the human pathogen *Pseudomonas aeruginosa* [13]. Taken together, these results suggest that in *Exaiptasia*, the recruitment of MyD88 and downstream signaling to activation of NF-κB is induced by Vp, and like in *Hydra* must involve different PRRs than prototypical TLR homologs. Based on these findings, we hypothesize that an ancestral TLR-to-NF-κB signaling pathway responsive to bacterial stimuli evolved before transmembrane TLRs in early metazoans.

### Potential TNFR signaling to inflammation and apoptosis

Another important aspect revealed by this study is the activation of cytokine-dependent signaling pathways in the innate immune response to Vp, two of which could lead to apoptosis and an inflammatory response. Indeed, the regulation of the ‘TNF-mediated signaling pathway’ was enriched at 3, 6 and 12 h post infection. One of these pathways likely starts with the activation of the TNFR1, which is the receptor for the proinflammatory cytokine TNFα and the cytotoxic protein lymphotoxin-alpha in humans. The Ep_TNFR1 DEC (GenBank: KXJ17455) is up-regulated 223.9 times after 1 h, then again at a lower level after 3, 6 and 12 h. Several TRAFs including TRAF2, − 5 and the MAP3K14 among other MAPK3 homologs, are potentially acting downstream of Ep_TNFR1 and are up-regulated at 6 and 12 h. Notably, more support for the TNFR1 pathway is shown with the TNFAIP3 and the TNIP1 homologs that are both up-regulated at 3, 6, 12 h. TNFAIP3 is an essential component of the ubiquitin-editing protein complex that ensures the transient nature of inflammatory signaling pathways, while TNIP1 inhibits the NF-κB activation and TNF-induced NF-κB-dependent gene expression by regulating the activity of TNFAIP3. Finally, the transcription factor AP-1 is up-regulated at each time point and may influence the immune response via activation of Ep_TNFR1.

The other cytokine-dependent pathway may involve the TNFRSF-mb16 (GenBank: KXJ29589) homologs, which contain a Death domain and are up-regulated at 3, 6 and 12 h. The Ep_TNFRSF-mb16 sequence resembles the Fas receptor (FasR or TNFRSF6 gene) which plays a central role upon Fas ligand (FasL) binding in the physiological regulation of programmed cell death in humans via its interaction with FADD and CASP8. In the coral *A. digitifera*, thirteen TNFR-like sequences containing the DD were identified and proposed as mediators of apoptosis through caspase activation [51]. Moreover, the conservation of the TNF-induced apoptotic response was demonstrated in the coral *Acropora digitifera* [52]. We also identified candidates potentially acting up- and down-stream of FasR, such as matrix metalloproteinase and FasLG homologs.

### Apoptosis as defense mechanism

In aiptasia’s response to infection, a functional enrichment for ‘regulation of apoptotic process’ could be detected at 3 h. The major cell death pathways, including apoptotic cell death, are a crucial barrier against microbial infection [53–55]. For instance, in response to bacterial infection, apoptosis or programmed cell death is used in the host innate immune response to: 1) eliminate pathogens at the early stage of infection without emitting alarm signals, and 2) induce dendritic cells (DCs) to engulf apoptotic bodies containing infected microbes [56]. Among the 216 DECs supporting the enrichment, many key players of apoptosis as well as genes with potential connections to the apoptotic pathway were identified at each time point. For example, the Ep_CASP8 is up-regulated at 3 and 6 h and likely functions within the cells. If interacting with the Ep_TNFRSF proteins, Ep_CASP8 would represent the extrinsic (receptor-mediated) pathway to apoptosis. On the other hand, the intrinsic (mitochondria-mediated) pathway is strongly supported by the up-regulation of ATF4, Bcl-2, Bcl-W, Bax, Bak, Apaf-1 as well as CASP9 homologs in Ep (Supplementary Table 4). Both pathways could engage the apoptotic executor - CASP3, which is up-regulated for the duration of the infection with a peak of 1250 in fold change at 6 h. These results strongly suggest that apoptosis plays an important role in the innate immune response of Ep against bacterial pathogens. As Ep is capable of recovering from a punctual exposure to Vp, it is probable that apoptosis plays a part in the removal of invading bacteria and the survival of the host. Further work will determine if and how Ep is capable of containing a bacterial invasion via programmed cell death.

### Comparison between responses to Vp infection and LPS

The genes of the TLR-to-NF-κB like pathway identified in the Ep’s response to Vp infection were not activated by LPS. *Hydra* polyps exposed to LPS treatments activate the expression of the antimicrobial peptides in a dose-dependent manner, suggesting that LPS is an inducer of immune defenses in cnidarians [35]. Indeed, studies on corals have shown that LPS can trigger an immune response in several species [28, 29, 57], and more specifically genes of the TLR-to-NF-κB pathway [34, 50]. However, LPS failed to stimulate an engineered *Hydra* LRR protein to activate NF-κB signaling in human cells, whereas flagellin did [35]. Similarly, LPS did not stimulate the sole *Nematostella* TLR to activate a reconstituted Nv-TLR-to-NF-κB pathway in 293 T cells, while flagellin and heat-inactivated Vc did [32]. Finally, in an attempt to induce innate immune response in *Exaiptasia*, Poole et al. [11] exposed anemones to LPS and peptidoglycan, but did not detect any significant changes in gene expression, and decided to use live *Serratia marcescens* instead. Interestingly, in this study the LPS-induced TNFα factor was up-regulated in response to both LPS and Vp. Overall, these results suggest that in cnidarians lacking TLR homologs, LPS can still trigger the production of cytokines and antimicrobial peptides independently of the TLR-to-NF-κB pathway.

### Novel genes responsive to infection

Several of the genes similar to the potential novel actinarian immune genes (NG1, NG2, and NG3) proposed by van der Burg et al. [45] based on domain architectures, are here annotated F-box only protein 11, 4 and F-box/LRR-repeat protein 14, and found up-regulated at all four time points. This study also reveals for the first time in a cnidarian that diverse G-protein coupled receptor (GPCR) homologs are highly regulated all along the infection and in response to LPS exposure (Supplementary Table 8). A variety of GPCRs are expressed in T cells and were recently linked to an important role in the mediation of immunity in humans [58–60]. Thus Ep_GPCRs constitute fascinating molecular targets towards the identification of ancestral immune cells in early-diverging metazoans and/or the activation of innate immunity via sensory pathways.

### Anthozoan innate immune response to pathogen infection

In the present study, we detected many more up-regulated DECs than down-regulated ones, but also the correlation between the stress intensity and the number of DECs was: the greater the stress over time, the greater the up-regulation (Table 1). Vidal-Dupiol et al. [61–63] have looked at the physiological and gene expression response of the coral *Pocillopora damicornis* (Pd) under temperature-dependent infection with Vc. Among the immune genes detected in Pd exposed to non-virulent Vc many also took part in this anemone’s response to Vp including NF-κB, AP-1, MyD88, and C3. However, these genes show the opposite regulation under the virulent Vc treatment, leading the authors to conclude the exact opposite to what was found in our study, “the greater the stress, the greater the down-regulation”. Therefore, besides the biological differences between anemones and corals, the confounding effects of temperature-induced bleaching and Vc’s virulence, the durational difference (i.e. days versus hours), as well as the various virulence mechanisms used by the different pathogens (Vc versus Vp), all contribute to the contradictory relationships found in Vidal-Dupiol et al. [63] and our experiment.

Heat shock proteins (HSPs) are also among the actively regulated genes during a significant portion of the onset of Vp infection (Supplementary Table 4), which suggests that they play a role in defending against pathogenic invasion. This supports the findings in an experiment to determine the capacity of Ep for immunological priming and memory, in which Brown and Rodriguez-Lanetty [43] used 2D fluorescent gel electrophoresis and mass spectrometry to detect differentially expressed HSPs in anemones exposed to sub-lethal Vc treatment for 4 weeks. Here, HSP70 and HSP90 DECs were up-regulated at 3, 6 and 12 h in the response to Vp (Supplementary Table 4). Higher HSP expression levels in primed anemones were suggested to be an evolutionary trait that helps anemones to resist successive encounters with pathogens as long as they occur within a six-week period [43] and were thought to be more likely relevant to immunological priming conditions than pathogen clearance. However, the transcriptional changes of HSP transcripts observed here also suggest a role in the early defense against pathogens.

Our data provide strong support for the involvement of complement and coagulation cascades, NOD-like receptor signaling, lysosome, and apoptosis throughout the 12 h of infection (Fig. 2; Supplementary Tables 6 and 7). These were also the pathways most affected in the responses of Ep to Vc detected using RNA-seq to explore the genetic links between anthozoan-algal symbiosis and innate immunity [27]. Similar to our results, more DECs were found up-regulated than down-regulated in response to 24 h exposure to Vc (i.e. 2155 versus 1552 DECs, respectively). Using KEGG pathway enrichment analysis, one pathway was significantly over-represented: the complement pathway under Vc treatment only. Our results build on Roesel and Vollmer’s [27] results by pointing to the functional equivalence to canonical PRRs in cnidarians as well as many potential downstream immune signaling genes.

## Conclusion

To our knowledge, this is the first time series study of the transcriptomic response to bacterial infection in a cnidarian model. Thousands of differentially expressed sequences were responsive to the infection over 12 h. Among the most interesting discoveries were indications that several innovations, including possible neofunctionalization, conservation of signaling pathways over conservation of particular key genes, and involvement of lineage-specific immunity genes, are represented in the innate immune response of aiptasia. Recognition of bacterial pathogens is likely fulfilled by putative novel immune receptors. Transduction of immune signals through a TLR-to-NF-κB like pathway is supported. Two TNFR pathways could possibly lead to an inflammatory response and apoptosis. And uncharacterized actinarian-specific genes with novel immune-domain architectures are responsive to bacterial infection. Altogether these results reveal the genes responsive to bacterial infection among innate immunity candidates in anemones, as well as potential novel players offering many new exciting research subjects to advance knowledge of innate immunity in early-diverging metazoans.

## Methods

### Experimental design

Individual clones of the sea anemone Ep strain CC7 were acquired from the Pringle laboratory at Stanford University and kept at the Scientific Center of Monaco under controlled conditions in the Ecosystems and Immunity Laboratory of the Biomedical Department. Anemones are maintained in three liter tanks inside a culture chamber (Percival Scientific, USA) at constant 27 °C with 20 μmol.m^− 2^.s^− 1^ of light (12:12 h). FSW at 0.45 μm is renewed every week and anemones are fed twice a week with artemias, except on the week of the experiment during which they fast.

For the infection experiment, a single anemone or biological replicate of approximately half a centimeter in diameter was placed in each well of eight 6-well plates filled with 10 ml of 0.22 μm FSW. Anemones were kept still overnight until fixed to the bottom.

Vp strain RIMD 2210633 serotype O3:K6 came from the laboratory of Prof. Kodama (Osaka University, Japan). Routinely, frozen Vp are cultured at 37 °C in Luria-Bertani liquid medium (LB) supplemented up to a final concentration of 1.5 g/L NaCl. For the infection treatment, Vp was then grown again at 27 **°**C in LB supplemented with NaCl to a final concentration of 3.5 g/L and without agitation for 24 h. The Vp cultures were centrifuged at 2500 rpm for 10 min, the bacterial pellet resuspended in 0.2 μm FSW to be concentrated 10 times, and 500 μL were dispensed into the wells containing the anemones in 9.5 ml of FSW. The final concentration of Vp was 10^8^ cfu/ml. Preliminary kinetics experiments using this concentration of pathogen were performed to choose relevant time points at which samples would be fixed for further molecular analyses. For the LPS treatment, ultrapure LPS from *E. coli* K12 (Invivogen cat. Tlrl-peklps) was added to reach a final concentration of 10 μg/ml in the medium containing the anemones.

After Vp or LPS added to treatment wells, anemones were placed in the Percival chamber at 27 **°**C under gentle orbital agitation of 500 rpm for the time of the experiment. The LPS treated samples were collected after 3, 6 and 12 h, whereas the Vp treatments were also sampled at 1 h. At each time point, whole anemones were detached with a beveled transfer pipette, transferred in 1.5 ml tubes and frozen in liquid nitrogen for further molecular analyses. Three independent anemones (*n* = 3) were collected at each time point for each condition to result in 33 samples total (Vp exposure: 3 biological replicates × 2 conditions × 4 time points and LSP: 3 biological replicates × 1 condition × 3 time points).

### Monitoring of physiological appearance

Photographs were taken using a stereomicroscope Stemi 305 trino (Zeiss) mounted with a digital camera 5MP Toupcam (Touptek). Photos were taken at each time point under bright light and the 0.8X magnification to monitor the overall appearance of the animals. Further observations were recorded at 5X magnification to assess the progression of the infection and the integrity of the animal tissue. Animals were considered dead when not contracting after being touched.

### RNA isolations

Frozen anemone tissues were disrupted and cells lysed in Trizol reagent (Ambion life technologies) to isolate RNA. To do so, approximately 100 uL of 1 mm diameter glass beads in 1.5 ml tubes filled with 1 ml of lysis buffer were used for anemones weighing approximately 100 mg. Tissue was completely disrupted using a Precellys 24 tissue lyser (Bertin Technologies) at 500 bpm for 2 × 5 s. Samples were then immediately placed on ice and total RNA extraction protocol was executed as follows: Two chloroform phase separations were performed on ice in order to precipitate the polysaccharides from the aqueous phase as much as possible. 300 uL of chloroform was added to the tissue lysate the first time, hand shaken for 15 s and settled at room temperature for 5 min, then centrifuged at 13000 g for 15 min at 4 **°**C. 200 uL of chloroform was added to the aqueous phase the second time and the previous steps repeated. The second aqueous phase was then added to an equal volume of chilled 100% ethanol and transferred onto a column for purification. The purification, including a DNase treatment step, followed the Direct-zol RNA MiniPrep Plus protocol (cat# R207 from Zymoresearch). RNA samples were then sent to Eurofins (Europe) for quality control on the Bioanalyzer (Agilent), cDNA library preparation using polyA selection (96 RNA-seq library from total RNA protocol, Illumina) and sequencing. cDNA libraries were sequenced as 2 × 100 bp paired-end reads on the Illumina HiSeq2500 with chemistry v4 and high-output run mode.

### Sequencing data processing

Raw sequences in fastq files were processed to remove Illumina adapters, low quality bases and short reads using the Trimmomatic-0.36 tool [64]. New reference transcriptomes were assembled de novo using all of the *Exaiptasia pallida* libraries produced here and either guided by the Ep genome produced by the Pringle Laboratory (version 1.1) or genome-free using Trinity v2.6.5 release [65]. Contiguous sequences or contigs were used to retrieve gene descriptions using BLASTX against the non-redundant NCBI database with the following filter: E-value < 10^− 3^, and only BLAST description annotation excluding the terms ‘unknown’, ‘hypothetical’, or ‘uncharacterized’. In addition, coding regions were predicted using TransDecoder and the predicted peptide sequences were used to retrieve further gene annotations from several different databases using BLASTP against Uniprot, Pfam, Swissprot and UniRef90. HMMER 3.1b2 (hmmer.org) and the Pfam (April 2020) database were also used to identify conserved protein domains present in our predicted peptide database. Depictions of domain architecture were based on conserved Domain Architecture Retrieval Tool (NCBI) results. Transmembrane domains were identified via the TMHMM (v.2.0) of the DTU Bioinformatics server. Contigs belonging to the anemone were isolated from the rest of sequences using BLASTX against the Ep predicted protein database produced by the Pringle Laboratory [10]. Transcript-level quantification was estimated for gene and isoform expression by allocating multi-mapping reads among all transcripts of the reference transcriptome using the RSEM [66] and Bowtie-2 [67] packages.

Two different gene expression analyses were performed on the count data: 1) a pairwise differential expression analysis (PDEA) comparing infected or LPS treated samples to controls using the ‘edgeR’ Bioconductor package [68], and 2) a time course expression analysis (TCEA) detecting genes with significant expression profile differences using the ‘maSigPro’ Bioconductor package [69], both implemented in the OmicsBox (Version 1.3.11 [70];) platform. Genes with low read counts across libraries (i.e. samples) were filtered out on a count-per-million basis equal to 1. The raw library sizes were normalized using the Trimmed mean of M values method from edgeR. For the PDEA, a simple design was used to compare treated versus control conditions at each time point. The Exact Test (in edgeR) with the strengthened estimation against outlier features option was used to detect differential expression. For the TCEA, a multiple series time course design was used to detect differentially expressed genes using a false discovery rate correction < 0.05 for variable selection in the stepwise regression. A cutoff value for the R-square of the regression model was set to 0.9. The optimal number of clusters (i.e. similar gene expression profiles) was determined using the model-based clustering method (in maSigPro 1.58.0). Results according to the selection procedure consisted in: 1) genes with differential expression at the first time point, 2) genes with a linear differential expression over time, 3) genes with a transitory differential expression, 4) genes with differential expression over time in controls, and 5) genes with differential expression profiles between treated and control samples.

Command lines used for the transcriptome assembly are provided in Supplementary File 1. The complete list of DECs containing sequence IDs, hits information, folds changes and statistical results is accessible in the Supplementary Tables 4 and 5. The tables of DECs in Supplementary Tables 6 and 7 show DECs with fold changes > 5 or < − 5, homolog peptide sequence similarity values > 70%.

### Functional analyses

#### Immune class GO categorization

To visualize the progress of aiptasia’s immune response to the infection, GO terms for differentially expressed contigs at each time point were categorized according to the ‘immune-class’ classification method by Zhi-Liang Hu [71]. Briefly, the CateGOrizer tool allows simplifying the visualization of functional annotations from gene lists by counting all paths between child and parent GO terms. Here, all paths between child and parent GO terms (using the cumulative count method: multiple paths possible between a single child and a single parent) were counted for the whole transcriptome as well as for the lists of DECs corresponding to each time point. The percentage of ‘immune_class’ GO term paths was then calculated as follows: GO path % = nbr. of GO paths in DECs list for GO:000xyz/nbr. of GO paths in transcriptome for GO:000xyz.

#### Targeted gene ontology

The GO codes corresponding to the parent terms ‘immune response’, ‘receptor activity’, ‘signal transduction’, and ‘peptidase activity’ were used to count all corresponding child terms within the DECs list for each time point. These DECs were then compared between each time point and visualized via Venn diagrams (http://bioinformatics.psb.ugent.be/webtools/Venn/; Supplementary Figure 3 A and B).

#### Enrichment analyses

The Fisher’s exact test (FET) was used to detect a significant enrichment for the immunity associated GO terms within the lists of DECs. The enrichment analyses were run using the OmicsBox platform [70] and the whole transcriptome annotated with GO terms as the reference gene set. FET were performed on the up- and down-regulated gensets produced via the PDEA at each time point. Results were then reduced to the most specific enriched GO categories applying a filter < 0.05 on FDR values to produce Fig. 2. The enriched GO categories discussed in this study were manually curated and represented by the genes provided in Supplementary Tables 3 and 4.

## Supplementary information

**Additional file 1 : Supplementary Figure 1**.

**Additional file 2 : Supplementary Table 1**.

**Additional file 3 : Supplementary Figure 2**.

**Additional file 4 : Supplementary Figure 3**.

**Additional file 5 : Supplementary Figure 4**.

**Additional file 6 : Supplementary Table 2**.

**Additional file 7 : Supplementary Table 3**.

**Additional file 8 : Supplementary Table 4**.

**Additional file 9 : Supplementary Table 5**.

**Additional file 10 : Supplementary Table 6**.

**Additional file 11 : Supplementary Table 7**.

**Additional file 12 : Supplementary Table 8**.

**Additional file 13 : Supplementary File 1**

## Data Availability

The datasets generated during the current study will be publicly available in the NCBI SRA repository under the BioProject accession number: PRJNA665134, on January 1st 2021.

## Abbreviations

Ep: *Exaiptasia pallida*
Vp: *Vibrio parahaemolyticus*
LPS: Lipopolysaccharides
PRRs: Pattern recognition receptors
NLRs: NOD-like receptors
RLRs: RIG-I-like receptors
CniFL: Cnidarian ficolin-like
TLRs: Toll-like receptors
Sm: *Serratia marcescens*
Vc: *Vibrio coralliilyticus*
LRR: Leucine-rich repeat
FSW: Filtered sea water
cfu: colony forming unit
GO: Gene ontology
DECs: Differentially expressed contigs
FDR: False discovery rate
FET: Fisher’s exact test
BP: Biological process
MF: Molecular function
CC: Cellular component
SR: Scavenger receptors
MDS: Multidimensional scaling
logFC: log Fold Change
PAMP: Pathogen-associated molecular pattern
TMD: Transmembrane domain
RBLs: Rhamnose-binding lectins
CLRs: C-type lectins
DCs: Dendritic cells
NGs: Novel genes
GPCRs: G protein-coupled receptors
Pd: *Pocillopora damicornis*
LB: Luria-Bertani liquid medium
PDEA: Pairwise differential expression analysis
TCEA: Time course expression analysis
