# Supplementary figures and images for: Gene expression kinetics of Exaiptasia pallida innate immune response to Vibrio parahaemolyticus infection
Source: BMC Genomics. 2020 Nov 9;21:768. doi: 10.1186/s12864-020-07140-6 (PMC7654579; doi:10.1186/s12864-020-07140-6)

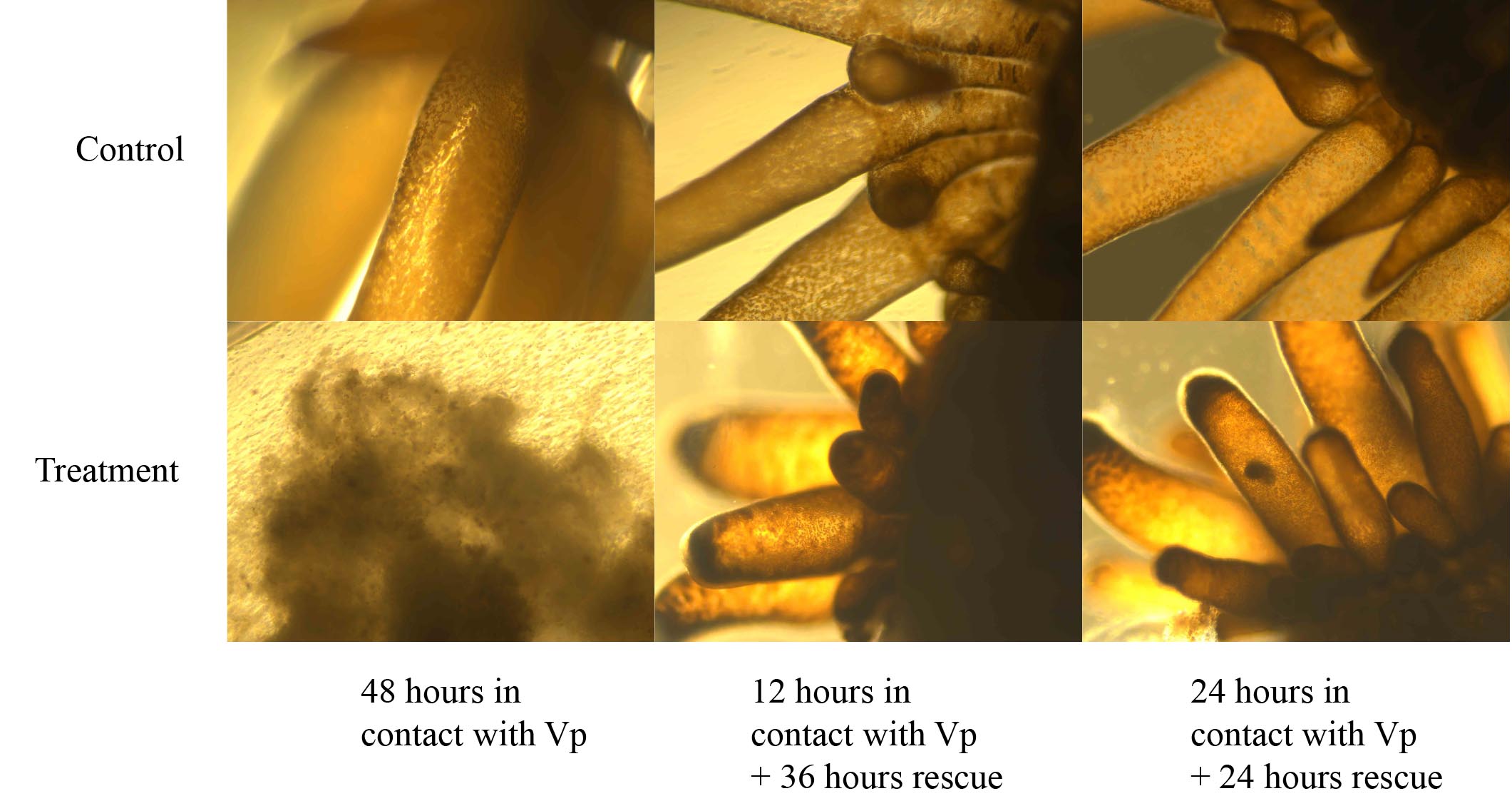

Supplement: Supplementary file 1 — Additional file 1 : Supplementary Figure 1. [file 12864_2020_7140_MOESM1_ESM.jpg]

Down-regulated at 1h

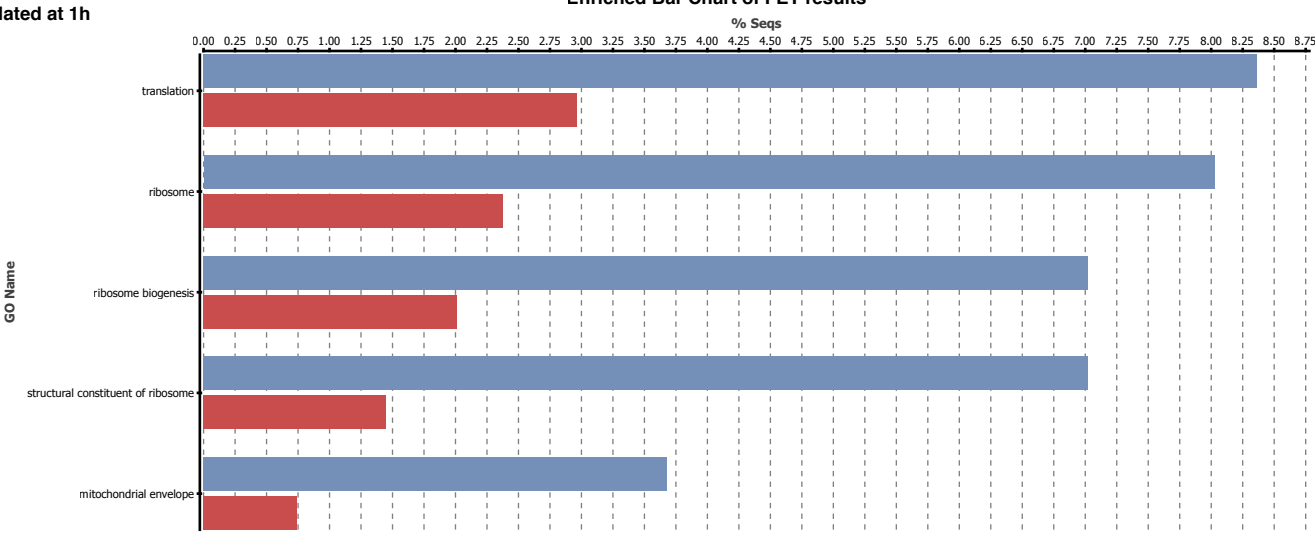

Down-regulated at 3h

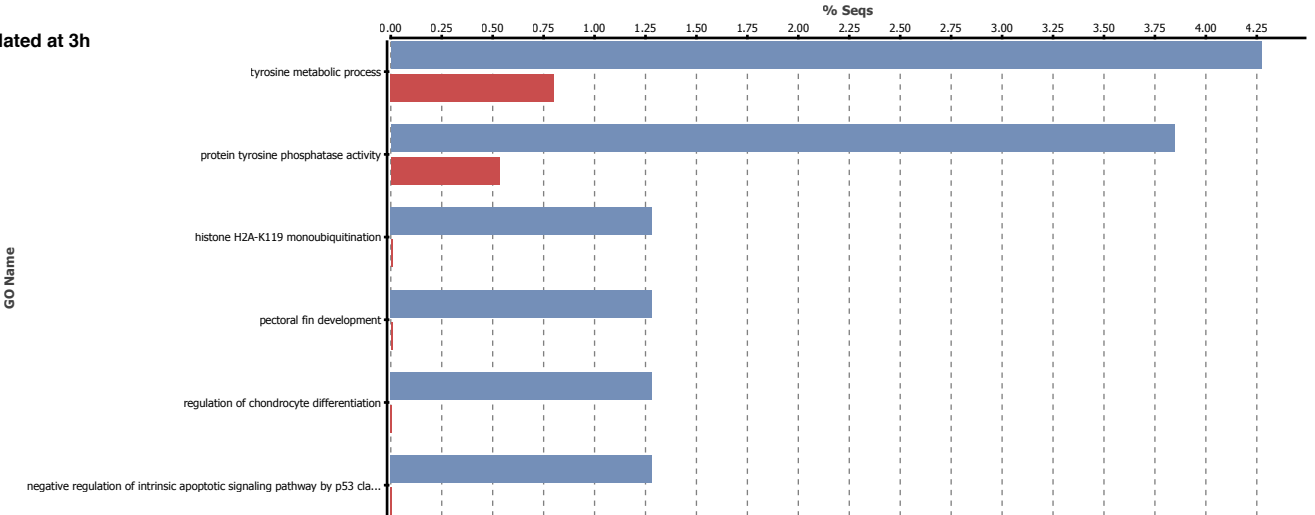

Down-regulated at 6h

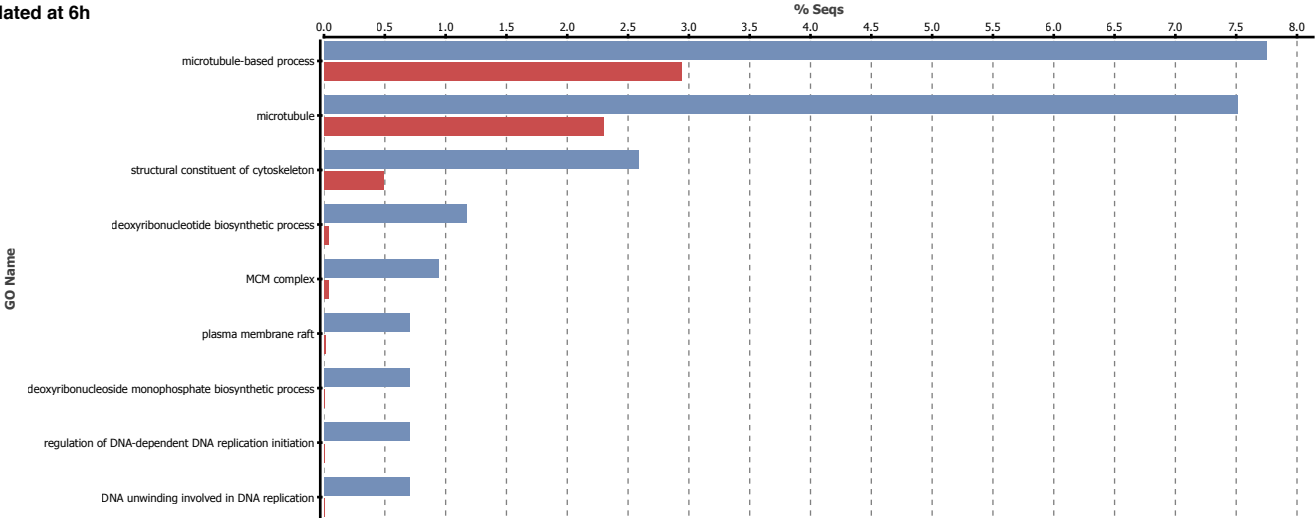

Down-regulated at 12h

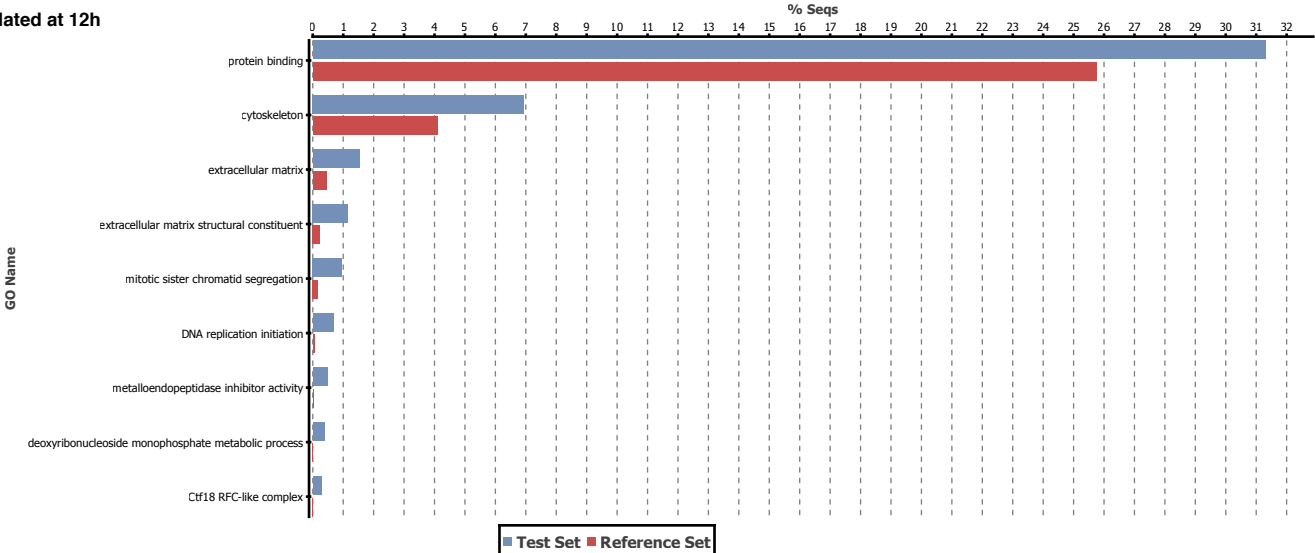

Supplement: Supplementary file 5 — Additional file 5 : Supplementary Figure 4. [file 12864_2020_7140_MOESM5_ESM.pdf]
