# Supplementary material for: Gene expression kinetics of Exaiptasia pallida innate immune response to Vibrio parahaemolyticus infection
Source: BMC Genomics. 2020 Nov 9;21:768. doi: 10.1186/s12864-020-07140-6 (PMC7654579; doi:10.1186/s12864-020-07140-6)
Supplement: Supplementary file 2 — Additional file 2 : Supplementary Table 1. [file 12864_2020_7140_MOESM2_ESM.pdf]

Create Count Table (Transcript-level) Results

Name: Ctl_vs_VopC+_cinetics_count_table

Input 1: Reference Transcript Sequences

132,179 sequences have been recovered from the fasta file 'Aiptasia_transcriptome_assembly_132179contigs_blastxfast_pringleprot_20200311_annotated.fasta'.

Transcripts	Min Length	Max Length	Avg Length
132,179	201	49,073	1,506.902

Input 2: FASTQ Files

A total of 23 samples have been processed.

File Name	Sample Name	Format	Sequencing
Aipt50_1.fastq.gz, Aipt50_2.fastq.gz	Aipt50	gz	Paired-End
Aipt60_1.fastq.gz, Aipt60_2.fastq.gz	Aipt60	gz	Paired-End
Aipt58_1.fastq.gz, Aipt58_2.fastq.gz	Aipt58	gz	Paired-End
Aipt47_1.fastq.gz, Aipt47_2.fastq.gz	Aipt47	gz	Paired-End
Aipt69_1.fastq.gz, Aipt69_2.fastq.gz	Aipt69	gz	Paired-End
Aipt57_1.fastq.gz, Aipt57_2.fastq.gz	Aipt57	gz	Paired-End
Aipt46_1.fastq.gz, Aipt46_2.fastq.gz	Aipt46	gz	Paired-End
Aipt68_1.fastq.gz, Aipt68_2.fastq.gz	Aipt68	gz	Paired-End
Aipt56_1.fastq.gz, Aipt56_2.fastq.gz	Aipt56	gz	Paired-End
Aipt67_1.fastq.gz, Aipt67_2.fastq.gz	Aipt67	gz	Paired-End
Aipt66_1.fastq.gz, Aipt66_2.fastq.gz	Aipt66	gz	Paired-End
Aipt65_1.fastq.gz, Aipt65_2.fastq.gz	Aipt65	gz	Paired-End
Aipt42_1.fastq.gz, Aipt42_2.fastq.gz	Aipt42	gz	Paired-End
Aipt64_1.fastq.gz, Aipt64_2.fastq.gz	Aipt64	gz	Paired-End
Aipt41_1.fastq.gz, Aipt41_2.fastq.gz	Aipt41	gz	Paired-End
Aipt51_1.fastq.gz, Aipt51_2.fastq.gz	Aipt51	gz	Paired-End
Aipt40_1.fastq.gz, Aipt40_2.fastq.gz	Aipt40	gz	Paired-End
Aipt39_1.fastq.gz, Aipt39_2.fastq.gz	Aipt39	gz	Paired-End
Aipt49_1.fastq.gz, Aipt49_2.fastq.gz	Aipt49	gz	Paired-End
Aipt38_1.fastq.gz, Aipt38_2.fastq.gz	Aipt38	gz	Paired-End
Aipt59_1.fastq.gz, Aipt59_2.fastq.gz	Aipt59	gz	Paired-End
Aipt48_1.fastq.gz, Aipt48_2.fastq.gz	Aipt48	gz	Paired-End
Aipt37_1.fastq.gz, Aipt37_2.fastq.gz	Aipt37	gz	Paired-End

Results Overview

Input Reads		Aligned Reads		Total	
Name	Total Records	Aligned 1 Time	Aligned Multiple Times	Overall Alignment Rate	Not Aligned
Aipt50	19,445,945	3,769,695 / 19.386%	8,350,665 / 42.943%	12,120,360 / 62.33%	7,325,585 / 37.672%
Aipt60	16,357,983	2,908,514 / 17.78%	6,566,558 / 40.143%	9,475,072 / 57.92%	6,882,911 / 42.077%
Aipt58	14,090,241	2,390,623 / 16.967%	5,617,524 / 39.868%	8,008,147 / 56.83%	6,082,094 / 43.165%
Aipt47	24,346,001	4,428,820 / 18.191%	8,865,378 / 36.414%	13,294,198 / 54.61%	11,051,803 / 45.395%
Aipt69	14,309,284	2,326,607 / 16.259%	5,142,401 / 35.938%	7,469,008 / 52.2%	6,840,276 / 47.803%
Aipt57	20,504,264	3,413,070 / 16.646%	7,472,510 / 36.444%	10,885,580 / 53.09%	9,618,684 / 46.911%
Aipt46	16,427,231	3,140,757 / 19.119%	6,268,348 / 38.158%	9,409,105 / 57.28%	7,018,126 / 42.723%
Aipt68	18,418,127	3,055,366 / 16.589%	6,743,549 / 36.614%	9,798,915 / 53.2%	8,619,212 / 46.797%
Aipt56	21,386,205	3,685,738 / 17.234%	7,585,815 / 35.471%	11,271,553 / 52.7%	10,114,652 / 47.295%
Aipt67	9,806,211	1,628,434 / 16.606%	3,864,019 / 39.404%	5,492,453 / 56.01%	4,313,758 / 43.99%
Aipt66	18,944,469	3,299,797 / 17.418%	6,977,198 / 36.83%	10,276,995 / 54.25%	8,667,474 / 45.752%
Aipt65	17,672,645	3,011,572 / 17.041%	6,176,964 / 34.952%	9,188,536 / 51.99%	8,484,109 / 48.007%
Aipt42	11,613,380	2,090,282 / 17.999%	4,212,253 / 36.271%	6,302,535 / 54.27%	5,310,845 / 45.73%
Aipt64	20,514,463	3,490,317 / 17.014%	7,377,556 / 35.963%	10,867,873 / 52.98%	9,646,590 / 47.023%
Aipt41	10,456,691	1,928,147 / 18.439%	3,826,138 / 36.59%	5,754,285 / 55.03%	4,702,406 / 44.97%
Aipt51	8,347,074	1,581,554 / 18.947%	3,381,660 / 40.513%	4,963,214 / 59.46%	3,383,860 / 40.539%
Aipt40	20,756,364	3,610,811 / 17.396%	7,664,714 / 36.927%	11,275,525 / 54.32%	9,480,839 / 45.677%
Aipt39	15,607,714	2,954,993 / 18.933%	5,611,535 / 35.954%	8,566,528 / 54.89%	7,041,186 / 45.113%
Aipt49	28,271,202	5,381,985 / 19.037%	11,684,154 / 41.329%	17,066,139 / 60.37%	11,205,063 / 39.634%
Aipt38	20,197,821	3,773,126 / 18.681%	7,185,573 / 35.576%	10,958,699 / 54.26%	9,239,122 / 45.743%
Aipt59	16,637,531	2,724,440 / 16.375%	6,507,603 / 39.114%	9,232,043 / 55.49%	7,405,488 / 44.511%
Aipt48	14,481,906	2,834,747 / 19.574%	5,503,363 / 38.002%	8,338,110 / 57.58%	6,143,796 / 42.424%
Aipt37	9,300,619	1,762,887 / 18.955%	3,369,264 / 36.226%	5,132,151 / 55.18%	4,168,468 / 44.819%

Warnings:

- 4,957 features (3.75%) for which no aligned reads was detected in any of the samples. Features with low counts provide little evidence for differential expression so they should be filtered out to improve further analysis.

Analysis Parameters

Parameter	Value
Sequencing Data	Paired-End Reads
Upstream Files Pattern	_1
Downstream Files Pattern	_2
Gene-level Estimations	false
Append Poly(A) Tails	false
Estimate RSPD	true
Strand Specificity	Strand Specific Reverse
Generate Alignment Files	true
Alignment Files	/Users/francois/Desktop/Aiptasia_project_iMac/Time_series_analyses

References

- Li B. and Dewey CN. (2011). RSEM: accurate transcript quantification from RNA-Seq data with or without a reference genome. *BMC bioinformatics, 12*, 323.
- Langmead B. and Salzberg SL. (2012). Fast gapped-read alignment with Bowtie 2. *Nature methods, 9*(4), 357-9.
- OmicsBox - Bioinformatics made easy. BioBam Bioinformatics (Version 1.3.11). March 3, 2019. www.biobam.com/omicsbox.
