# Supplementary material for: Gene expression kinetics of Exaiptasia pallida innate immune response to Vibrio parahaemolyticus infection
Source: BMC Genomics. 2020 Nov 9;21:768. doi: 10.1186/s12864-020-07140-6 (PMC7654579; doi:10.1186/s12864-020-07140-6)

Proportions of accumulative counts for Immune Class GO terms of the significantly regulated genes over those present in the transcriptome

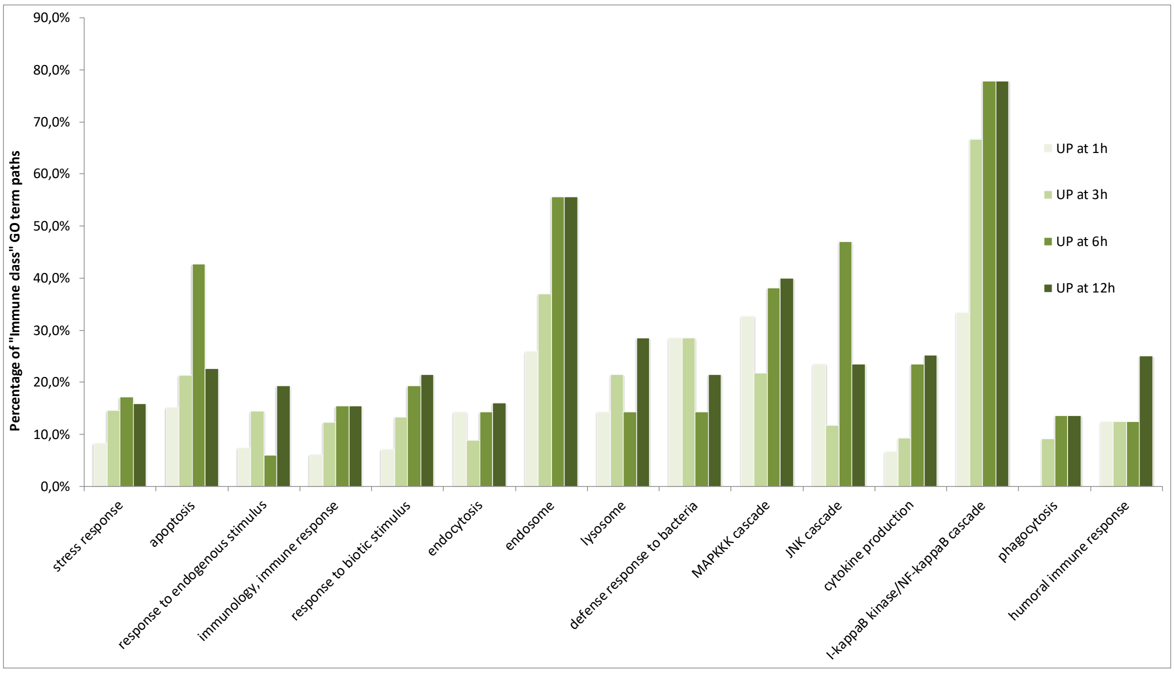

Supplement: Supplementary file 3 — Additional file 3 : Supplementary Figure 2. [file 12864_2020_7140_MOESM3_ESM.pdf]
