# Supplementary material for: Gene expression kinetics of Exaiptasia pallida innate immune response to Vibrio parahaemolyticus infection
Source: BMC Genomics. 2020 Nov 9;21:768. doi: 10.1186/s12864-020-07140-6 (PMC7654579; doi:10.1186/s12864-020-07140-6)
Supplement: Supplementary file 4 — Additional file 4 : Supplementary Figure 3. [file 12864_2020_7140_MOESM4_ESM.zip › Supp_FIG3_A_Venn_diagram_series_UP_regulated_panelA.pdf]

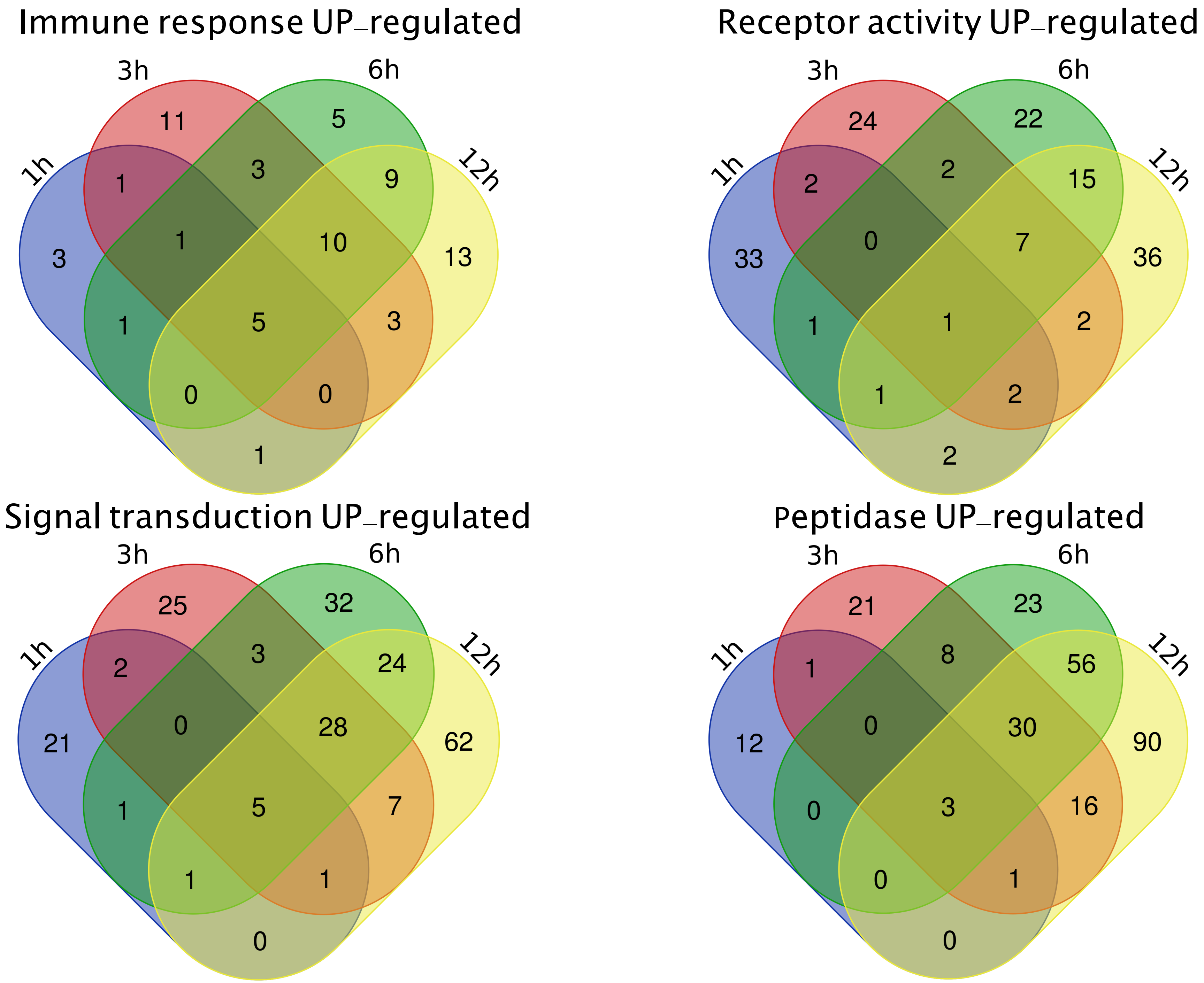
Immune response UP_regulated
3h
6h
1h
12h
11
5
1
3
9
3
1
10
13
1
5
3
0
0
1
Receptor activity UP_regulated
3h
6h
1h
12h
24
22
2
2
15
33
0
7
36
1
1
2
1
2
2
Signal transduction UP_regulated
3h
6h
1h
12h
25
32
2
3
24
21
0
28
62
1
5
7
1
1
0
Peptidase UP_regulated
3h
6h
1h
12h
21
23
1
8
56
12
0
30
90
0
3
16
0
1
0
