# Supplementary material for: Gene expression kinetics of Exaiptasia pallida innate immune response to Vibrio parahaemolyticus infection
Source: BMC Genomics. 2020 Nov 9;21:768. doi: 10.1186/s12864-020-07140-6 (PMC7654579; doi:10.1186/s12864-020-07140-6)
Supplement: Supplementary file 4 — Additional file 4 : Supplementary Figure 3. [file 12864_2020_7140_MOESM4_ESM.zip › Supp_FIG3_B_Venn_diagram_series_DOWN_regulated_panelB.pdf]

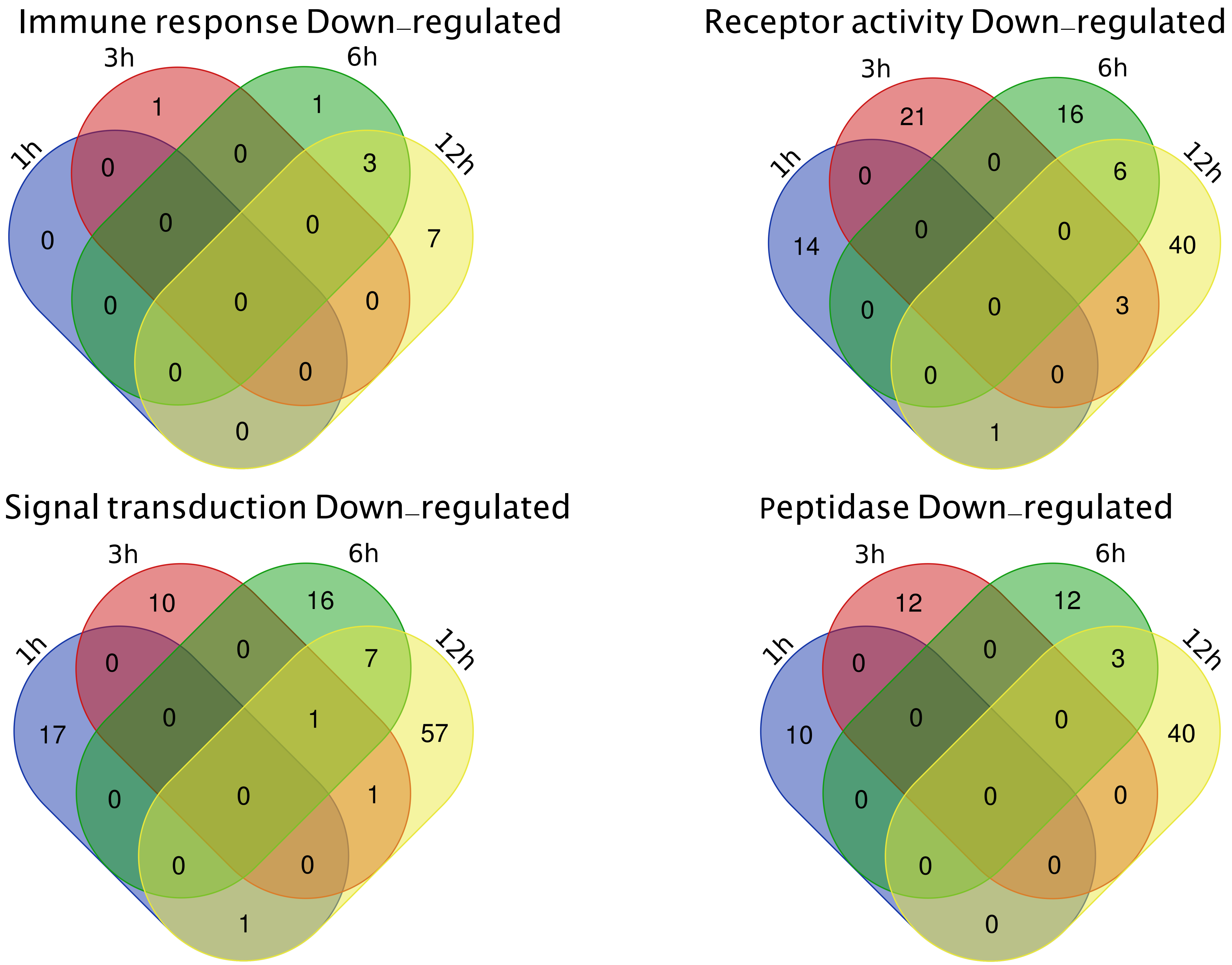
Immune response Down_regulated
1h
3h
6h
12h
0
1
1
7
0
0
3
0
0
0
0
0
0
0
0
Receptor activity Down_regulated
1h
3h
6h
12h
14
21
16
40
0
0
6
0
0
3
0
0
0
0
1
Signal transduction Down_regulated
1h
3h
6h
12h
17
10
16
57
0
0
7
0
1
0
0
1
0
0
1
Peptidase Down_regulated
1h
3h
6h
12h
10
12
12
40
0
0
3
0
0
0
0
0
0
0
0
