# Supplementary material for: Gene expression kinetics of Exaiptasia pallida innate immune response to Vibrio parahaemolyticus infection
Source: BMC Genomics. 2020 Nov 9;21:768. doi: 10.1186/s12864-020-07140-6 (PMC7654579; doi:10.1186/s12864-020-07140-6)
Supplement: Supplementary file 10 — Additional file 10 : Supplementary Table 6. [file 12864_2020_7140_MOESM10_ESM.pdf]

Contig code	Category and gene name	Time	Fold change (Treatment/Control)	FDR	Length (bp)	e-value	Similarity %
	**Complement and coagulation cascades**						
TRINITY_DN99618_c4_g1_i3	Coagulation factor VIII	3h	15,0	5,74E-03	1923	0,00E+00	74,1
TRINITY_DN94556_c0_g2_i3	Coagulation factor VIII	3h	-5,9	3,73E-04	2167	0,00E+00	71,8
TRINITY_DN102340_c0_g2_i7	Coagulation factor VIII	3h	-98,1	7,26E-04	1998	0,00E+00	77,1
TRINITY_DN94633_c4_g3_i3	Coagulation factor V	6h	406,2	3,78E-03	520	6,64E-04	100,0
TRINITY_DN96168_c0_g1_i18	Coagulation factor VIII	6h	5,4	3,94E-02	1849	0,00E+00	71,5
TRINITY_DN95841_c1_g2_i11	Coagulation factor VIII	12h	21,9	1,72E-05	2005	9,22E-146	71,8
TRINITY_DN100716_c0_g1_i10	Coagulation factor VIII	12h	16,2	4,38E-03	1543	0,00E+00	79,9
TRINITY_DN102175_c1_g1_i1	Coagulation factor VIII	12h	-5,1	2,81E-04	985	6,15E-09	95,2
TRINITY_DN101526_c1_g2_i1	complement C2-like	12h	7,1	1,25E-02	1250	0,00E+00	71,9
	**Pattern recognition receptor**						
TRINITY_DN102253_c5_g1_i14	Lectin BRA-3	6h	11,1	3,01E-02	1327	1,31E-97	83,0
TRINITY_DN94240_c2_g1_i2	L-rhamnose-binding lectin CSL1	6h	5,6	7,91E-06	1377	1,07E-08	74,4
TRINITY_DN99643_c2_g1_i1	Galectin-3-binding protein	12h	37,2	8,68E-03	1967	1,51E-21	91,7
TRINITY_DN98679_c2_g4_i2	galectin-3-binding protein B-like	12h	13,4	4,16E-02	502	2,85E-14	75,9
TRINITY_DN102716_c6_g1_i4	L-rhamnose-binding lectin CSL1	12h	-50,6	2,26E-04	481	8,31E-08	70,5
TRINITY_DN101085_c1_g2_i7	Protein NLRC5	1h	-7,8	3,24E-02	492	8,47E-90	77,0
TRINITY_DN99262_c1_g2_i8	Protein NLRC5	3h	-99,2	4,21E-02	1062	0,00E+00	74,3
TRINITY_DN97902_c0_g2_i2	Protein NLRC5	6h	6,4	1,09E-02	1294	5,59E-123	95,8
TRINITY_DN102593_c7_g2_i3	protein NLRC5-like	6h	-7,4	1,98E-04	857	5,53E-06	100,0
TRINITY_DN97902_c0_g2_i3	Protein NLRC5	12h	102,8	2,15E-02	1560	0,00E+00	84,2
TRINITY_DN98730_c2_g1_i6	Protein NLRC5	12h	6,2	8,95E-05	1715	3,10E-129	72,7
TRINITY_DN97224_c0_g1_i6	Protein NLRC5	12h	5,1	4,00E-03	426	3,31E-84	86,4
TRINITY_DN102591_c5_g1_i5	Protein NLRC5	12h	-5,8	2,32E-05	1842	0,00E+00	71,8
TRINITY_DN101085_c0_g2_i1	Protein NLRC5	12h	-6,6	3,74E-03	600	7,99E-101	70,7
TRINITY_DN102591_c5_g1_i6	Protein NLRC5	12h	-12,0	3,01E-02	1278	2,28E-156	74,4
TRINITY_DN101180_c2_g2_i3	macrophage scavenger receptor types I and II-like	6h	10,0	1,78E-03	1629	3,00E-132	89,9
TRINITY_DN96991_c1_g1_i28	Scavenger receptor cysteine-rich type 1 protein M130	6h	-5,4	3,14E-02	1125	1,35E-103	90,5
TRINITY_DN96991_c1_g1_i16	Scavenger receptor cysteine-rich type 1 protein M130	6h	-215,4	9,70E-03	2145	6,90E-51	100,0
TRINITY_DN101180_c2_g2_i3	macrophage scavenger receptor types I and II-like	12h	14,1	3,84E-02	1629	3,00E-132	89,9
TRINITY_DN94702_c1_g5_i2	macrophage scavenger receptor types I and II-like	12h	5,0	1,15E-02	516	7,05E-82	92,6
TRINITY_DN101180_c2_g2_i12	macrophage scavenger receptor types I and II-like	12h	-19,5	6,09E-03	1476	5,39E-133	78,4
TRINITY_DN96991_c1_g1_i16	Scavenger receptor cysteine-rich type 1 protein M130	12h	-116,1	2,93E-02	2145	6,90E-51	100,0
	**Cytokines and regulators**						
TRINITY_DN95963_c0_g2_i6	interferon regulatory factor 1-like	1h	795,7	6,52E-03	6582	3,03E-47	74,5
TRINITY_DN95963_c0_g2_i2	interferon regulatory factor 1-like	1h	-98,1	4,09E-02	6589	3,04E-47	74,5
TRINITY_DN91014_c0_g1_i5	interferon regulatory factor 2-like isoform X1	3h	220,6	3,62E-02	1802	2,66E-106	72,1
TRINITY_DN95963_c0_g2_i8	interferon regulatory factor 1-like	3h	34,0	1,13E-06	6331	2,91E-47	74,5
TRINITY_DN98455_c5_g1_i5	stimulator of interferon genes protein-like	6h	18,9	2,52E-02	314	9,28E-62	81,2
TRINITY_DN100778_c2_g1_i8	Interferon-induced helicase C domain-containing protein 1	6h	12,7	2,04E-02	563	5,83E-13	97,0
TRINITY_DN98923_c0_g2_i2	Interferon-induced very large GTPase 1	6h	11,0	6,28E-05	1786	0,00E+00	74,5
TRINITY_DN101621_c0_g1_i11	Interferon-induced protein 44	6h	7,6	1,55E-03	2943	1,41E-118	92,4
TRINITY_DN90397_c0_g1_i1	interferon regulatory factor 1-like	6h	6,8	2,77E-03	1083	2,75E-08	100,0
TRINITY_DN100778_c2_g1_i15	Interferon-induced helicase C domain-containing protein 1	6h	6,5	3,30E-02	380	8,47E-14	97,0
TRINITY_DN98455_c5_g1_i9	stimulator of interferon genes protein-like	12h	171,5	4,12E-03	309	6,58E-68	84,4
TRINITY_DN93683_c0_g3_i2	interferon-induced helicase C domain-containing protein 1-like	12h	21,0	1,67E-02	2564	1,87E-07	96,6
TRINITY_DN90397_c0_g1_i1	interferon regulatory factor 1-like	12h	11,9	1,40E-06	1083	2,75E-08	100,0
TRINITY_DN98923_c0_g2_i2	Interferon-induced very large GTPase 1	12h	7,6	2,55E-05	1786	0,00E+00	74,5
TRINITY_DN93683_c0_g3_i4	interferon-induced helicase C domain-containing protein 1-like	12h	7,5	1,46E-02	3603	5,43E-06	96,5
TRINITY_DN95742_c0_g2_i1	gamma-interferon-inducible lysosomal thiol reductase-like	12h	5,5	2,73E-03	1297	2,32E-161	76,4
	**Adaptors and signal transducers**						
TRINITY_DN96193_c2_g1_i20	TNF receptor-associated factor 4	1h	6,5	2,24E-02	829	1,76E-174	80,0
TRINITY_DN96349_c4_g5_i8	TNF receptor-associated factor 2-like	1h	5,7	1,94E-02	4567	1,69E-20	91,2
TRINITY_DN101626_c4_g1_i2	TNF receptor-associated factor 3-like	3h	39,4	4,34E-03	2361	0,00E+00	71,2
TRINITY_DN101626_c4_g1_i14	TNF receptor-associated factor 3-like	3h	5,1	1,19E-06	624	5,91E-127	88,0
TRINITY_DN102242_c4_g1_i2	TNF receptor-associated factor 6-like	3h	5,1	1,57E-02	1065	2,70E-107	80,3
TRINITY_DN96200_c0_g3_i2	TNF receptor-associated factor family protein DDB_G0290931-like	3h	-9,9	4,80E-03	1164	4,00E-10	96,9
TRINITY_DN94744_c1_g1_i13	TNF receptor-associated factor 5-like	3h	-230,6	1,79E-02	4522	9,49E-28	73,8
TRINITY_DN97184_c0_g1_i4	TNF receptor-associated factor 4	6h	488,2	1,04E-03	3082	0,00E+00	79,9
TRINITY_DN94744_c1_g1_i1	TNF receptor-associated factor 5-like	6h	10,3	2,72E-02	386	5,45E-81	71,0
TRINITY_DN98792_c6_g4_i4	TNF receptor-associated factor 5-like	6h	6,2	4,83E-02	310	1,75E-22	74,2
TRINITY_DN99330_c1_g1_i14	TNF receptor-associated factor 6-like	6h	5,7	5,97E-05	2940	5,22E-45	90,3
TRINITY_DN96200_c0_g3_i1	TNF receptor-associated factor family protein DDB_G0290931-like	6h	-21,1	2,72E-05	794	6,31E-25	98,7
TRINITY_DN99497_c9_g1_i1	TNF receptor-associated factor family protein DDB_G0290931-like	6h	-21,6	3,07E-02	232	1,82E-06	100,0
TRINITY_DN101308_c0_g1_i11	TNF receptor-associated factor 3-like	12h	13,8	7,35E-03	1231	5,35E-118	72,4
TRINITY_DN102242_c4_g1_i2	TNF receptor-associated factor 6-like	12h	8,7	2,04E-03	1065	2,70E-107	80,3
TRINITY_DN101626_c4_g1_i8	TNF receptor-associated factor 5-like	12h	6,6	1,28E-03	1968	1,01E-23	82,5
TRINITY_DN94744_c1_g1_i1	TNF receptor-associated factor 5-like	12h	6,3	6,47E-03	386	5,45E-81	71,0
TRINITY_DN101308_c1_g1_i2	TNF receptor-associated factor 3-like	12h	5,9	8,30E-07	938	4,28E-112	91,5
TRINITY_DN98792_c6_g4_i4	TNF receptor-associated factor 5-like	12h	5,8	4,63E-04	310	1,75E-22	74,2
TRINITY_DN99497_c9_g1_i1	TNF receptor-associated factor family protein DDB_G0290931-like	12h	-5,4	7,84E-04	232	1,82E-06	100,0
TRINITY_DN96200_c0_g3_i1	TNF receptor-associated factor family protein DDB_G0290931-like	12h	-6,5	6,89E-09	794	6,31E-25	98,7
TRINITY_DN101855_c1_g1_i5	tumor necrosis factor receptor superfamily member 1A-like	1h	223,9	2,06E-02	1835	0,00E+00	74,0
TRINITY_DN101855_c1_g1_i4	tumor necrosis factor receptor superfamily member 1A-like	6h	6,8	8,05E-04	1836	0,00E+00	74,0
TRINITY_DN98782_c1_g1_i1	tumor necrosis factor ligand superfamily member 6-like	12h	12,3	3,34E-03	1792	1,74E-34	98,5
TRINITY_DN99763_c1_g6_i1	Tumor necrosis factor ligand superfamily member 10	12h	6,4	1,20E-03	3579	3,14E-34	97,8
TRINITY_DN95157_c1_g1_i2	MyD88 protein	6h	254,7	1,40E-02	2302	3,03E-63	76,7
