# Supplementary material for: Gene expression kinetics of Exaiptasia pallida innate immune response to Vibrio parahaemolyticus infection
Source: BMC Genomics. 2020 Nov 9;21:768. doi: 10.1186/s12864-020-07140-6 (PMC7654579; doi:10.1186/s12864-020-07140-6)
Supplement: Supplementary file 11 — Additional file 11 : Supplementary Table 7. [file 12864_2020_7140_MOESM11_ESM.pdf]

Contig code	Gene name	Time	Fold change	FDR	Length (bp)	e-value	Similarity %
	**Apoptosis cascades**						
TRINITY_DN98251_c2_g1_i5	Acidic leucine-rich nuclear phosphoprotein 32 family member A	1h	635,0	3,03E-09	2263	1,02E-96	89,2
TRINITY_DN101855_c1_g1_i5	Tumor necrosis factor receptor superfamily member 1A-like	1h	223,9	2,06E-02	1835	0,00E+00	74,0
TRINITY_DN97148_c0_g1_i9	Serine/threonine-protein kinase PAK 3-like isoform X1	1h	19,1	1,33E-02	6474	0,00E+00	91,6
TRINITY_DN94576_c6_g3_i1	Apoptosis regulator R1-like	1h	12,2	2,39E-02	1171	6,44E-28	87,6
TRINITY_DN98251_c2_g1_i4	Acidic leucine-rich nuclear phosphoprotein 32 family member A	1h	-265,4	6,78E-05	2525	7,19E-96	89,2
TRINITY_DN94876_c0_g1_i1	Serine/threonine-protein kinase 10	1h	-677,6	1,01E-04	1506	2,91E-77	78,9
TRINITY_DN99936_c3_g1_i16	V-ets erythroblastosis virus E26 oncogene homolog 1 (avian)	3h	249,4	3,68E-05	4620	2,13E-36	76,4
TRINITY_DN98251_c2_g1_i6	Acidic leucine-rich nuclear phosphoprotein 32 family member A	3h	174,8	4,10E-03	2379	2,49E-96	89,2
TRINITY_DN101626_c4_g1_i2	TNF receptor-associated factor 3-like	3h	39,4	4,34E-03	2361	0,00E+00	71,2
TRINITY_DN96791_c0_g4_i1	Bcl-2 homologous antagonist/killer-like	3h	5,7	2,63E-06	2220	3,57E-120	80,0
TRINITY_DN99266_c2_g2_i8	E3 ubiquitin-protein ligase RING2-like	3h	-6,0	1,97E-02	2273	0,00E+00	76,9
TRINITY_DN94256_c3_g1_i14	Caspase 3-like protein	6h	1250,5	3,06E-17	2773	0,00E+00	79,4
TRINITY_DN97184_c0_g1_i4	TNF receptor-associated factor 4	6h	488,2	1,04E-03	3082	0,00E+00	79,9
TRINITY_DN95231_c4_g1_i12	Nbn1 subunit of the NuA4 histone acetyltransferase complex	6h	405,7	5,78E-05	726	1,84E-36	72,4
TRINITY_DN98936_c0_g1_i14	Mitogen-activated protein kinase kinase kinase NPK1	6h	14,2	3,29E-02	1719	0,00E+00	72,6
TRINITY_DN94256_c3_g1_i10	Caspase 3-like protein	6h	12,7	4,05E-02	2724	0,00E+00	79,6
TRINITY_DN101855_c1_g1_i4	Tumor necrosis factor receptor superfamily member 1A-like	6h	6,8	8,05E-04	1836	0,00E+00	74,0
TRINITY_DN98458_c1_g1_i3	Apoptotic protease-activating factor 1-like	6h	5,3	6,02E-07	3985	0,00E+00	81,8
TRINITY_DN97323_c1_g1_i3	3-phosphoinositide-dependent protein kinase 1	6h	-332,0	1,93E-02	1721	0,00E+00	77,9
TRINITY_DN102651_c3_g2_i1	Calcium/calmodulin-dependent protein kinase type 1D	12h	157,2	4,56E-02	2503	0,00E+00	86,9
TRINITY_DN97943_c0_g1_i3	E3 ubiquitin-protein ligase MIB1-like isoform X1	12h	88,0	4,36E-02	3281	0,00E+00	74,4
TRINITY_DN94256_c3_g1_i10	Caspase 3-like protein	12h	40,9	3,17E-02	2724	0,00E+00	79,6
TRINITY_DN100515_c2_g5_i6	Bcl-2-like protein 1	12h	21,7	1,73E-02	3688	7,87E-90	71,3
TRINITY_DN97148_c0_g1_i3	Serine/threonine-protein kinase PAK 3-like isoform X1	12h	6,8	2,73E-02	6433	0,00E+00	91,8
TRINITY_DN101308_c1_g1_i2	TNF receptor-associated factor 3-like	12h	5,9	8,30E-07	938	4,28E-112	91,5
TRINITY_DN91689_c0_g1_i4	Dual specificity protein kinase Ttk	12h	5,7	6,86E-08	1018	5,26E-71	93,4
TRINITY_DN97087_c0_g1_i14	T-box transcription factor TBX1	12h	-11,9	1,05E-03	1103	7,35E-133	85,5
